# Mapping taste and flavour traits to genetic markers in lettuce *Lactuca sativa*

**DOI:** 10.1016/j.fochms.2024.100215

**Published:** 2024-08-23

**Authors:** Martin Chadwick, Jonathan R. Swann, Frances Gawthrop, Richard Michelmore, Davide Scaglione, Maria Jose-Truco, Carol Wagstaff

**Affiliations:** a Department of Food and Nutritional Sciences, University of Reading, Harry Nursten Building, Whiteknights, Reading RG6 6DZ, UK; b Faculty of Medicine, University of Southampton, University Road, Southampton SO17 1BJ, UK; c Tozer Seeds Pyports, Downside Bridge Rd, Cobham KT11 3EH, UK; d UC Davis Genome Center, 451 Health Sciences Drive, Davis CA 95616, USA; e IGA Technology Services, Via J. Linussio, 51 Z.I.U.Udine, 33100, Italy

**Keywords:** Sesquiterpene, Lactuca sativa, NMR, Quantitative trait loci, Breeding, Flavour

## Abstract

Lettuce is the most highly consumed raw leafy vegetable crop eaten worldwide, making it nutritionally important in spite of its comparatively low nutrient density in relation to other vegetables. However, the perception of bitterness caused by high levels of sesquiterpenoid lactones and comparatively low levels of sweet tasting sugars limits palatability. To assess variation in nutritional and taste-related metabolites we assessed 104 members of a *Lactuca sativa* cv. Salinas x *L. serriola* (accession UC96US23) mapping population. Plants were grown in three distinct environments, and untargeted NMR and HPLC were used as a rapid chemotyping method, from which 63 unique Quantitative Trait Loci (QTL) were identified. We were able to identify putative regulatory candidate genes underlying the QTL for fructose on linkage group 9, which accounted for up to 36 % of our population variation, and which was stable across all three growing environments; and for 15-p-hydroxyyphenylacetyllactucin-8-sulfate on linkage group 5 which has previously been identified for its low bitterness, while retaining anti-herbivory field effects. We also identified a candidate gene for flavonoid 3′,5′- hydroxylase underlying a polyphenol QTL on linkage group 5, and two further candidate genes in sugar biosynthesis on linkage groups 2 and 5. Collectively these candidate genes and their associated markers can inform a route for plant breeders to improve the palatability and nutritional value of lettuce in their breeding programmes.

## Highlights

• NMR spectroscopy is an effective and rapid alternative to chromatographic methods.
• Stable QTL were identified for sugars and bitter sesquiterpene lactones.
• We identify breeding markers for flavour and nutritional traits.
• Candidate genes are identified for key metabolites.

## 1 Introduction

### 1.1 Introduction

Lettuce is an important Asteraceous leafy vegetable crop consumed across the world, and in the western diet is one of the main constituents of salads by weight. It is a source of folates, polyphenols, terpenoids, dietary fibre and has a low glycemic index (GI) (Shi et al., 2022). These factors make it one of the most commonly consumed vegetables (Statistica, 2023) associated with leading a healthy lifestyle. The absolute concentration of secondary metabolites is lower compared to other leafy vegetables, particularly brassicas and spinach, however the large quantities consumed make it rate highly in total contribution of polyphenols to western diets (Song et al., 2010).

Major traits of consumer interest that determine the acceptability and potential market size of a crop are taste, flavour and health benefits. These traits are determined by the concentrations of sugars and beneficial secondary metabolites, such as polyphenols, sesquiterpenoids, and fatty acids. These metabolites can affect consumer liking and buying preference (Chadwick et al., 2016), seen in the trend toward consuming food considered healthy, additionally driving up consumption of lettuce and increasing the consumption of beneficial compounds.

Sesquiterpenes are a significant class of plant secondary metabolites which are characteristic of the Asteraceae and have evolved as phytoalexins and anti-feedants in lettuce to protect the crop against chewing insect pests. In lettuce these are predominantly sesquiterpene lactones (STLs); more than 2 % of the plant’s dry weight can be made up of these compounds (Heinrich et al., 1998). Lettuce is a perceived as being bitter by many consumers; sensitivity to bitterness is particularly prominent in younger children, who represent a major target group for healthy eating, and for whom fruit and vegetable consumption is lacking (Saksvig et al., 2005, Cooke, 2007, Rodrigues et al., 2020). STLs are the primary source of bitterness in lettuce, which are detected by HTAS2R receptors (Brockhoff et al., 2007). A range of health benefits have been reported for STLs isolated from across the plant kingdom, primarily from the Asteraceae, and include those present in lettuce (Bischoff et al., 2004, Zhang et al., 2016, García et al., 2020, Wang et al., 2020). The health benefits, which include anticancer and anti-inflammatory effects, antifungal, anxiolytic, analgesic and antitrypanosomal activities (Moujir et al., 2020), are broadly attributed to the presence of an α-methyl-γ-lactone (αMγL) functional group (Kupchan et al., 1971, Macías et al., 1996, Hehner et al., 1998, Chadwick et al., 2013, Schomburg et al., 2013, Kemboi et al., 2022) and an unsaturated carbonyl moiety (Chen et al., 1998, Lyß et al., 1998, Siedle et al., 2004) as a part of the lactone ring structure.

In addition to sesquiterpene lactones, phenolics represent another major nutritional group in lettuce. Quercetin, luteolin, chlorogenic acid, chicoric acid, caftaric acid and coumaric acid and their derivatives are present in lettuce and both profile and abundance are highly variable between varieties. Up to 2 mg.g^−1^ (fresh weight) of polyphenolics has been reported (Heimler et al., 2012) and considered as a major component of the health giving properties of plants (Llorach et al., 2004, García-Macías et al., 2007, Fraga et al., 2019, Catalkaya et al., 2020). Their taste is slightly bitter and astringent, though with less of an impact on overall bitterness than STLs.

Concentration of sugars counterbalances bitterness (Green et al., 2010). Sugar content of food is an international concern in terms of contributing to diet-related disease when consumed in high quantities, particularly within processed foods (Machado et al., 2020). Nonetheless, there is a strong consumer preference for sweeter and less bitter lettuce (Chadwick et al., 2016). The health benefits of consuming leafy vegetables, such as fibre and secondary metabolites are considered to outweigh the negative impacts of consuming sugars at the concentrations they are found in lettuce (∼50 nmol.kg^−1^ fresh weight; (Gent, 2012)). Sugars remain an important breeding target to generate new vegetable varieties with good consumer acceptability.

In order understand the environmental impact on sesquiterpene metabolism and to search for stable QTL which were minimally affected by the growing environment we used a mapping population derived from *L. sativa* cv. Salinas x *L. serriola* UC96US23. The population of 104 F_9_ lines was grown in three environments: an indoor controlled environment with limited stress; and two field-based trials simulating commercial practice but one with minimal soil nitrogen and with soil nitrogen provided above concentrations found in common commercial practice.

### 1.2 Nuclear Magnetic Resonance (NMR) spectroscopy as a rapid tool in determining the metabolome of lettuce

NMR spectroscopy enables the simultaneous measurement of a large range of molecules from a variety of biochemical classes in rapid and reproducible manner. The high-throughput nature of NMR makes it an ideal tool for metabolomic profiling allows the biomolecular fingerprints of large sample sets to be reliably assessed in a relatively short time window. Previous work has characterised the metabolomes of lettuce leaves by ^1^H and ^13^C NMR (Sobolev et al., 2005) and comparisons drawn between similar varieties (Sobolev et al., 2010, Lanzotti et al., 2022). In addition, ^1^H NMR spectroscopy has been applied to identify differences in genotype, and the impact of environmental conditions on the metabolome of various crops, including tomato (Mattoo et al., 2006, Schauer et al., 2006), bell pepper (Villa-Ruano et al., 2018) maize (Piccioni et al., 2009) and tea (Rubel Mozumder et al., 2020), and shown to have utility in distinguishing critical developmental and physiological differences in a wide variety of foods (Mannina et al., 2012). This approach has the advantage of minimal required sample processing, reducing the degradation of unstable molecules, and permitting a broad range of structurally diverse compounds to be assessed within a single sample, broadening an untargeted approach to metabolomic studies.

The metabolic pathways for synthesis of sugars, polyphenols, and sesquiterpenoids are well studied, however, it is currently not known which stages in metabolism, catabolism, translocation, or utilisation have the greatest overall effect on metabolite concentration in lettuce at a commercially relevant developmental stage. We used a wide cross of *L. sativa* cv. Salinas (a commercial variety) and *L. serriola* accession UC96US23 (a wild accession) a mapping population shown to have high variation in flavour and nutritional components. To establish stability in different commercially relevant environments, we explored matched a conventional field trial with standard fertiliser application with a lower nitrogen trial to simulate reduced fertiliser inputs, and a controlled environment to simulate indoor farming. In this study, QTL analysis has been used to identify genomic regions that have the greatest influence on quantitative metabolite traits. Subsequent finemapping to refine the locus can be utilised to elucidate the genetic basis underlying the trait (Korstanje and Paigen, 2002).

## 2 Methods

### 2.1 Plant growth, and processing

F_9_ recombinant inbred lines (RILs) were supplied by the Michelmore lab (Genome Center, UC Davis, USA) and 102 RILs plus their parents, *L. sativa* cv. Salinas and the wild *L. serriola* UC96US23, were propagated by Tozer Seeds (Cobham, UK). For these studies, one trial of the whole population was grown in a controlled environment rooms (Weiss Gallenkampf, Loughbrough UK) at The University of Reading. Temperatures were kept at a constant 25 °C, 16 h day length, with light level of 170 µmol.m^2−1.^s^−1^ and humidity of 60 %. Plants were harvested after 49 days, at a mature, commercially viable, stage prior to floral transition. Six plants of each line were grown from which four representative plants were sampled to account for and remove lost and non-representative plants.

Two field trials (high N and residual (low) N) were grown at the trials site (Tozer Seeds, Cobham, UK) in four randomised blocks for each treatment; high nitrogen plots were fertilised to 180 kg N.Ha^−1^, while low nitrogen was the residual levels available on the plot, which averaged 50 kg N.Ha^−1^. The residual N plots represent the minimum recommended by RB209 fertiliser guide (MAFF, 2000), while the high N plot represents an over-fertilisation of around 50 kg N.Ha^−1^ compared to the accepted maximum value recommended for growers. Six identical plants were grown per plot, and four most representative plants of each genotype were harvested. Field trials were harvested after 39 days.

For all trials all aerial parts were immediately flash frozen in liquid N, and subsequently lyophilised and milled to a powder at Rothamsted Research UK. Four plants from each plot were homogenised, and each of the four blocks from each environment were treated as an independent replicate for further analysis.

### 2.2 ^1^H NMR analysis

Lettuce leaves were lyophilised and 20 mg of this material was suspended into 1 ml of NMR buffer (containing 0.05 % of the internal standard trimethylsilypropionate [TSP]; w/v 80:20 D_2_O:CD_3_OD) (Sigma-Aldrich MO, USA) and mixed thoroughly. The solution was heated to 50 °C for 10 min in a waterbath, centrifuged at 6,000 x *g* for 5 min, and the supernatant was collected. The supernatant was subsequently heated to 90 °C for 2 min in a waterbath and rapidly cooled. Before analysis the samples were centrifuged for 5 min at 15 500 x *g* and the supernatant was assessed once by Bruker 700 MHz spectrometer (MA, USA) equipped with a cryoprobe for enhanced sensitivity. For each sample, a one-dimensional ^1^H NMR spectrum was acquired using a standard pulse sequence with water peak suppression (recycle delay-90° -t1 -90° -tm -90° -acquisition), 90° pulse of 12 µs. The recycle delay was set at 2 s and 100 ms mixing time. For each lettuce sample, 128 scans were collected after 8 dummy scans into 64,000 data points. A spectral width of 20 ppm was used and acquisition time per scan was 3.41 s. Each homogenised lettuce sample was analysed once, and were resampled if data collection failed.

All spectra were calibrated to the internal standard TSP at *δ* 0.00 ppm, and baseline and phase distortions were manually corrected using Topspin 2.1 software (Bruker, MA, USA). In-house spectral libraries, online resources, and previously published data was used to assign discriminatory peaks arising from the statistical models and metabolites of interest were identified by measuring the ^1^H NMR spectra of purified standards where available (Sessa et al., 2000, Sobolev et al., 2005, Wishart et al., 2013). A complete list of identified peaks is provided in Supplementary Table S1. Redundant signals arising from the water and TSP in the samples were excised from all spectra. The remaining spectra were manually aligned, normalised using a total area approach, and peaks of interest were integrated using in-house Matlab (MA, USA) scripts. In addition, 2D NMR experiments were performed on the parent lines from the controlled environment trial to inform metabolite identification (^1^H-COSY, ^1^H-TOCSY and ^1^H-DIPSI) using the same instrument and conditions as reported above. Whie this analytical method limits cannot accurately give absolute concentration of metabolites, the relative values are as powerful for QTL analyses.

This processed spectral data was used to construct principal components analysis (PCA) models and orthogonal projection to latent structures-discriminant analysis (OPLS-DA) models in Matlab using in-house scripts. For the OPLS-DA model, lettuce species (*L. sativa* v *L. serriola*) was used as the response vector and the predictive performance (Q^2^Y) of the model was assessed using a seven-fold cross-validation approach. The significance of this predictive performance was evaluated using a permutation approach (1,000 permutations, *P*<0.05). For the OPLS-DA model, the covariance of each spectral datapoint with class (*L. sativa* v *L. serriola*) are plotted and the colour indicates those datapoints that are significantly associated with class (red, *P*<0.05; black not significant).

### 2.3 Isolation of sesquiterpenes for use as standards

Sesquiterpenes which could not be accessed commercially were isolated chromatographically from *L. serriola*, to provide reference spectra for NMR spectroscopy. Spectral data for STLs is shown in Fig. S1. Plants were grown under glasshouse conditions at The University of Reading (UK) until initiating floral transition, when they were cut on alternate days and the latex harvested over two weeks. The extracts were prepared by suspending 1 ml of latex into 10 ml of ethyl acetate (SLS, Nottingham, UK), vortexing to mix, and centrifuging at 6 000 x g for 5 min to form a pellet. The supernatant was dried and resuspended into 1 ml of DMSO. Samples were separated by Prep-HPLC using 100 % MeOH solvent, using a phenomenex ‘Prodigy’ 5 µm ODS-3 100 Å, 250 x 21.2 mm column (Phenomenex CA, USA), and fractions collected manually according to the retention time of the peaks previously identified by HPLC-MS. All peaks were subsequently run by HPLC MS as described in Chadwick, Gawthrop *et. al* (2016) to confirm the identity and purity of the sesquiterpenoid standards. These were dried completely and resuspended in 1 ml of NMR solution (0.05 % TSP w/v 80:20 D2O:CD3OD) to allow for comparison with sample data.

### 2.4 QTL analysis

MapQTL 6 (Kyazma, Netherlands) (van Ooijen, 2011) was used to conduct QTL analysis in all cases. A linkage map generated by the Michelmore lab (UC Davis, USA; (Truco et al., 2013) was used to map the QTL. Predicted means were calculated for QTL mapping using Reduced Maximum Likelihood (REML). Logarithm of odds (LOD) threshold was determined for each trait by permutation testing (1000 permutations) to determine a threshold of statistical significance (p = 0.05). QTL were initially detected by interval mapping (IM) using a regression model. Significant markers were selected and used to inform automatic cofactor selection. Cofactors were taken into account for a final multiple QTL mapping (MQM) to confirm QTLs.

### 2.5 Candidate gene identification

Candidate genes were identified by consultation of KEGG (Kyoto Encyclopaedia of Genes and Genomes) metabolic pathways for sesquiterpenes, phenolics and sugars. Gene sequences encoding the transcription of these enzymes were taken from *Arabidopsis thaliana,* where possible, otherwise closest phylogenetic relative to lettuce was used. In some instances, sequences from fungi and microbes were explored where no plant relative could be found with the equivalent enzyme.

### 2.6 Mapping of synthesis genes

In an automated procedure using Perl script all sequences were BLASTed against the full draft *L. sativa* sequence (Lsat.1.v3.ScaffoldSequence) as the reference sequence, and aligned to the best matches using Exonerate aligner (Slater and Birney, 2005). Sequences were returned if they met the following stringency tests; 10 % of the full sequence, minimum allele frequency of 30 %, maximum rate of missing data of 30 %, minimum rate of segregation of 30 % and introns of less than 3000 bp. The maximum number of results returned per sequence was limited to the five strongest matches. Where several candidate gene identities aligned to the same sequence, the sequence was tentatively identified as the candidate gene with the strongest alignment. SNPs were identified within the mapping population by consulting sequence data for the RILs, and the markers were aligned to the existing genetic map using JoinMap 4 (Kyazma, Netherlands).

## 3 Results

### 3.1 NMR as a means of profiling the metabolome

From 285 independent NMR signals, 36 compounds were identified (Supplementary Table S1). Identified compounds of interest included amino acids, sugars, fatty acids, and polyphenols. Patterns of compound concentration were consistent with data from previous investigations using NMR and chromatographic methods (Sobolev et al., 2005).

Sesquiterpene lactones standards were isolated from lettuce midrib latex and purified by HPLC. Lactucin, lactucopicrin, and 8-deoxylactucin-15-oxalate were all identified and verified by mass spectrometry (Supplementary Fig. S1). Standards were individually assessed by NMR spectroscopy. Despite being distinct in isolation, common structural backbones meant they could not be individually identified in a complex sample, with the exception of 15*-p-*hydroxyphenylacetyllactucin-15-oxalate. For this reason, all other STL data reported were collected identified and quantified by HPLC-MS.

### 3.2 Variation in metabolite composition between species and growing environments

**Fig. 1:**
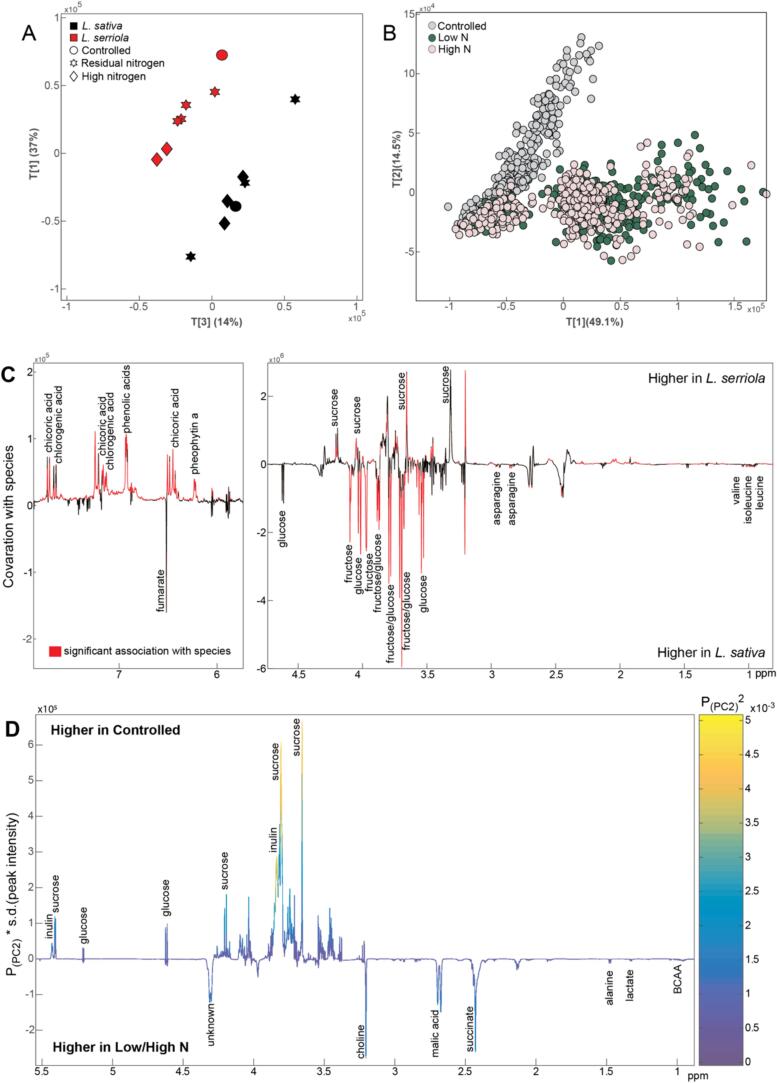
Biochemical variation in the ^1^H NMR spectral profiles *of L. serriola* and *L. sativa****.*** A) Scores plot (PC1 v PC3) from the principal components analysis (PCA) model comparing the species grown under controlled, high nitrogen and residual nitrogen conditions. B) Scores plot (PC1 v PC2) from the PCA model comparing all offspring grown under controlled, high nitrogen and residual nitrogen conditions. C) Orthogonal projection to latent structures-discriminant analysis (OPLS-DA) model comparing the metabolic profiles of *L. serriola* and *L. sativa* grown under all conditions (Q^2^Y=0.69; *p* = 0.001). Coefficient plot indicates how metabolites covary with species with red peaks indicating those that are significantly associated with species (p < 0.05). D) Loadings plot highlighting the metabolic features contributing to the PC1 scores in the PCA model comparing offspring from different growing conditions. (For interpretation of the references to colour in this figure legend, the reader is referred to the web version of this article.)

The parents of the mapping population, *L. sativa* cv. Salinas and *L. serriola* UC96US23, were selected to capture a wide interspecific genetic variation and to include a commercially relevant genotype. From the PCA model constructed on the full spectral profiles of both genotypes grown in different conditions, clear species-dependent metabolic variation was observed. Interestingly, genotype-specific variation was greater than that dependent on growing conditions, predominantly seen in separation along PC1 (capturing 37 % of variation) (Fig. 1A). As species-dependent variation was greater than environmental-induced variation, an OPLS-DA model was constructed on the *L. sativa* (*n* = 7) and *L. serriola* (*n* = 7) profiles grown in all conditions. A significant model with strong predictive performance (Q^2^Y=0.69; *P*=0.001; Fig. 1C) was obtained. This showed that *L. serriola* contained greater amounts of sucrose, chlorogenic acid, chicoric acid, and pheophytin a, while *L. sativa* contained greater amounts of glucose, fructose, asparagine, and the branched chain amino acids, valine, leucine and isoleucine.

**Fig. 2:**
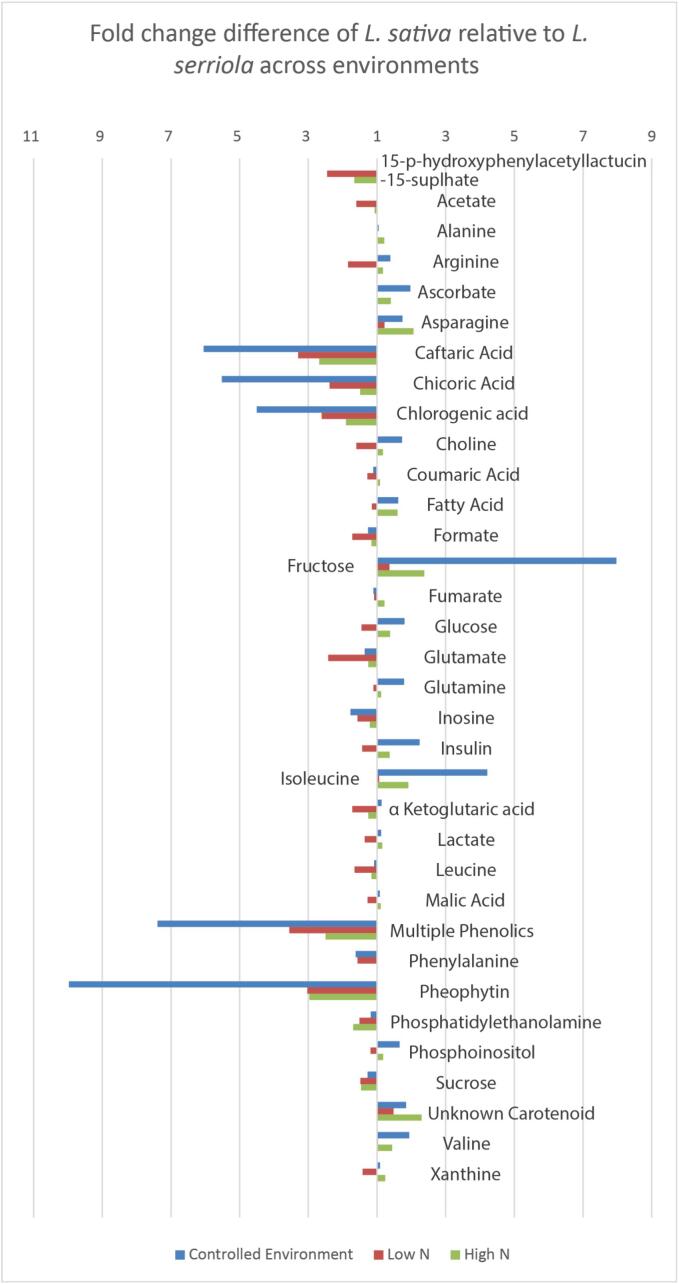
Percentage of *L. serriola* compound concentration relative to L. sativa in each tested environment***.*** Differences in concentration of each compound identified in each *L. sativa* and *L. serriola* in each experimental condition. Plants showed most extreme phenotypes in the controlled environment, with up to ten times difference in metabolite concentration (Pheophytin 9.9 times more concentrated in *L. serriola*). *L. serriola* consistently contained more polyphenols than *L. sativa*. Bars to the right of centre represent higher concentration of metabolite *in L. sativa* relative *to L. serriola*, bars to the left of the centre show lower concentrations of metabolites in *L. sativa* relative *to L. serriola*. Scores for each parental genotype was derived from averages of all NMR run peak areas.

Metabolite integrals extracted from the NMR data were compared to investigate the impact of growing conditions across the species. This difference between the genotypes is substantially less under field conditions than in the controlled environment trial (Fig. 2), but statistically significant differences remain between the parental genotypes, particularly, 1.35 (*p* = 0.03) and 2.38 (*p* = 0.021) fold higher fructose concentrations for *L. sativa* observed in the low and high nitrogen trials respectively. In low nitrogen conditions *L. serriola* accumulated more glucose than *L. sativa*, and lower fructose and glucose.

**Fig. 3:**
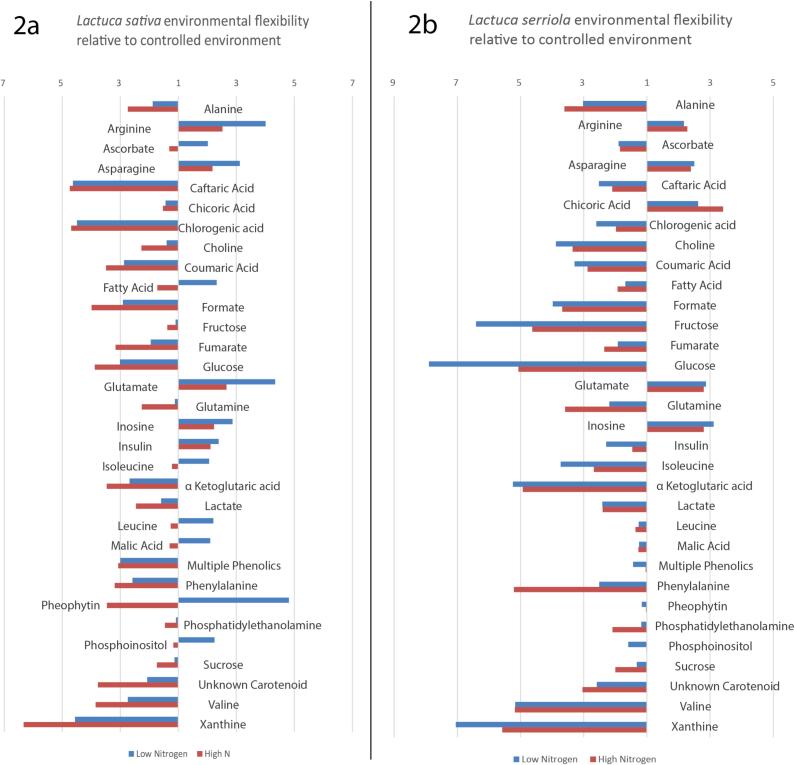
Fold change in selected compounds between the controlled environment and the two respective field trials for each of the parents***.*** Scores for each parental genotype was derived from averages of all NMR run peak areas. Shown is the fold change of each compound identified in each the low (residual) nitrogen trial and the high nitrogen trial, relative to the controlled environment, for each genotype. Bars to the right of centre represent higher concentration of metabolite in the field trial relative to the controlled environment, bars to the left of the centre show lower concentrations of metabolites in the field trial relative to the controlled environment.

Sucrose was found to be the most abundant sugar in all environments and the two field environments led to greater accumulation of sucrose, fructose, and glucose in both species compared to controlled environment (Fig. 3). Fructose accumulated at 7.97 fold higher concentration in *L. sativa* under controlled conditions compared to the wild species.

*L. serriola* had higher concentrations of phenolics than *L. sativa* (Fig. 1A, 1C; 5–8 ppm), particularly caftaric acid (2.6–6 fold, chicoric acid (1.5–5 fold) and chlorogenic acid (1.9–4.5 fold), which are the most abundant phenolic acids in lettuce. Caftaric acid accumulated up to 6-fold greater concentrations in the wild parent compared to the domesticated cultivar. Chicoric acid levels were highest in the controlled environment, for *L. serriola*, but only 25 % as high in the controlled environment for *L. sativa* compared to the two field trials. Caffeic acid, o-coumaric acid and chlorogenic acid levels were highest in the two field trials. Phenolic concentrations were amongst the most variable of the molecules assessed, RIL 11 is shown to be an outlier with high levels of chicoric, chlorogenic and caftaric acid, though RIL 103 had a higher total content of phenolics based on the common peak at 6.92 ppm. RIL 54 was consistently containing the lowest levels of these compounds. *L. serriola* was consistently higher than *L. sativa* in the case of all phenolic acids analysed (Supplementary Table S3).

Concentration of organic acids in the two parent types was similar; malic acid remained stable across all conditions, formic acid and fumaric acid concentrations increased within the field trials relative to the controlled environment (CE), with the greatest environmental influence coming from high nitrogen conditions (Fig. 3). Pheophytin (a form of chlorophyll *a*) was nearly 10 fold higher in *L. serriola* than its domesticated relative under controlled conditions, and around three times higher in field conditions which is consistent with the darker green colour observed in the wild species. However, whereas *L. sativa* increased pheophytin accumulation in the field compared to controlled environment *L. serriola* did not, perhaps indicating an adaptive capacity of the former to respond to light conditions that enable more photosynthesis to take place.

A total of 11 essential amino acids were identified (Table 1, Table 2). The largest differences were observed for isoleucine, which was 4.2 x higher in *L. sativa*, and glutamate, which was up to 2.42 x higher in *L. serriola,* under low nitrogen conditions compared to the controlled environment. Overall, total amino acid content was similar between the genotypes, indicating differences in profile arise from alternate pathways of protein synthesis rather than the overall capacity of the system. Amino acids glutamine and alanine along with choline were amongst the least variable molecules assessed, though only in the controlled environment (Supplementary Table S3).

**Table 1:** Selected QTLs detected by MQM mapping for all metabolic traits in the RIL mapping population of three environments***.*** Peak ppm gives the precise peak isolated for these QTL from a number of potential candidates. Linkage group represents the chromosome number to which the QTL corresponds. C3A is part of chromosome 3, but where the two parts cannot currently be linked by existing markers. All distances (Marker Position and QTL interval) are given in cM. QTL interval is the area in which LOD score is within 2 of the peak value, and represents the extent to which we can be confident to find a QTL. LOD is Log of the odds score. Variance indicates the percentage of phenotypic variation within the population can be explained at by that QTL. Additive effect indicates which parental allele causes positive change in trait value. Positive values indicate that the domesticated parental allele increased trait value while negative values indicate that the wild type allele increases the trait value.

**Peak Identity**	**Peak ppm**	**Linkage Group**	**Nearest Marker**	**Marker Position (cM)**	**QTL Interval (cM) ⱡ**	**LOD**	**% Variance Explained**	**Additive Effect**
**Controlled Environment Trial**								
valine	0.982	C1	CBVJ_0	54.24	5.203	3.86	17.8	0.022
isoleucine	1.014	C1	CBVJ_0	54.24	5.782	3.8	17.5	0.044
inulin	5.43	C1	CBVJ_0	54.24	6.203	3.31	15.4	−0.061
oleic acid	1.295	C1	ARCO_0	131.096	11.587	4.09	15.8	−0.017
phenylalanine	7.438	C2	AZKZ_0	37.152	6.348	3.56	13.9	−0.019
purine derivative*	7.803	C3	BIRI_0	2.881	3.862	5.38	21.4	−0.034
phenylalanine*	7.346	C3	AVSQ_0	18.607	5.392	3.63	16.8	−0.025
o-coumaric acid*	7.373	C3	AVSQ_0	18.607	5.392	3.78	17.4	−0.013
8-deoxylactucin-15-oxalate	(HPLC)	C3A	AXWN_0	7.94	4.93	2.84	23.4	−36.720
15-p-hydroxyphenylacetyllactucin-8-sulphate	(HPLC)	C4	ANFS_0	66.414	4.487	3.96	31.1	−1.919
glucose	3.5	C4	AYKH_0	192.777	7.327	3.42	15.9	0.018
glucose	3.23	C4	BNSR_0	226.24	6.799	3.89	17.9	0.049
SQDG	3.43	C4	ASQI_0	229.266	5.08	3.68	17	0.023
chicoric acid	6.486	C4	AOWD_0	254.897	15.661	4.99	15.6	−0.047
15-p-hydroxyphenylacetyllactucin-8-sulphate	6.835	C5	BDIA_0	155.250	4.521	3.550	14.800	−0.059
chlorogenic acid	2.13	C5	AJVD_0	242.661	6.849	3.58	16.5	−0.058
leucine	1.75	C5	AZLB_0	246.393	7.681	3.09	12.9	−0.016
chlorogenic acid	2.05	C5	AZLB_0	246.393	7.681	3.88	15.2	−0.010
fructose	3.543	C6	BHQG_0	151.451	5.944	4.47	12.8	0.020
purine derivative*	7.803	C7	AAUV_0	44.864	9.441	3.26	12.4	−0.025
glutamine	2.45	C7	BXPY_0	144.684	7.659	4.36	19.8	−0.036
aspartate	2.794	C7	BXPY_0	144.684	6.666	4.47	20.3	−0.019
chlorogenic acid	5.285	C8	AAOY_0	62.416	2.979	3.69	17	−0.019
oleic acid	5.353	C8	AFYL_0	63.987	6.524	3.03	12.2	−0.014
chlorogenic acid	2.05	C8	BQDJ_0	88.118	5.35	3.61	14.1	−0.010
unknown amino acid*	8.192	C8	AJTS_0	90.954	7.389	3.36	15.4	−0.019
cytidine	7.874	C9	AIZU_2	0.00	1.855	3.35	15.6	0.016
fructose	3.569	C9	ANAJ_0	6.687	4.005	3.48	16.2	0.015
sucrose and fructose	3.573	C9	ANAJ_0	6.687	4.005	3.17	14.8	0.015
chlorogenic acid	6.429	C9	ATFM_0	143.589	7.192	7.14	30.3	−0.044
chicoric acid	6.516	C9	ATFM_0	143.589	7.35	7.13	30.3	−0.032
sucrose and fructose	3.543	C9	ADPE_1	185.782	3.796	10.29	34.5	0.032
fructose	4.0	C9	ADPE_1	185.782	3.796	8.89	36.2	0.086

**High Nitrogen Trial**								
inulin*	4.064	C2	AHWV_0	110.122	21.009	3.66	14.9	−0.039529
sucrose	5.406	C2	BTNV_0	175.211		3.53	14.7	−0.058957
glucose	3.861	C4	AGWB_0	164.142	7.61	3.54	14.8	−0.038101
sucrose	4.193	C4	AGWB_0	164.142	7.934	3.76	13.6	−0.046087
glucose	3.853	C5	BDIA_0	155.25	7.61	3.32	13.9	−0.044652
glutamate	2.411	C7	BQEM_0	89.435	8.741	3.47	14.5	−0.054889
glutamine	2.451	C7	BXPY_0	144.684	7.659	3.93	16.3	−0.071828
amine/nucleic acid*	2.934	C9	BATF_0	16.38	6.23	5.59	22.3	−0.110971
fructose	4.098	C9	BKCQ_0	187.303	1.54	5.62	22.4	0.061337

**Low Nitrogen Trial**								
glutamate	2.388	C3	ARDR_0	55.815	5.49	6.1	24.1	−0.094601
arginine*	3.631	C4	BIRC_0	277.695	2.382	3.71	15.4	0.049693
glucose	3.401	C5	BDIA_0	155.25	6.083	3.82	15.9	−0.057939
sucrose	5.406	C6	AQNZ_0	136.693	4.404	3.74	15.5	0.063076
glucose	3.217	C7	CCMM_0	17.181	8.688	3.21	13.5	0.052116
amine/nucleic acid*	2.856	C9	BUEX_0	10.35	3.269	5.81	23.1	−0.130226
fructose	3.971	C9	AGID_0	183.763	7.739	5.28	18.3	0.053179
fructose	4.014	C9	BKCQ_0	187.303	1.54	9.25	29.2	0.069403

* Not confirmed								
ⱡ 2 LOD region								

fructose	3.971	C7	BVWO_0	2.891		3.42	11.3	0.042
fructose	4.014	C7	BCVF_0	24.544		4.29	12	0.044

fructose	3.971	C7	BVWO_0	2.891		3.42	11.3	0.042
fructose	4.014	C7	BCVF_0	24.544		4.29	12	0.044

**Table 2:** QTL which map to candidate genes***.*** Candidate genes sequences from KEGG pathways were added to the existing marker map. 5 QTL co-located to these candidate genes when they were remapped. Map distances changed due to the mapping algorithm used when updating the marker maps, so for comparison, the flanking markers in the original marker map are included. In each case the QTL which maps to the candidate gene also maps to the corresponding markers in the original map.

**Assigned Protein Name**	**E.C. identifier**	**TAIR accession (Arabidopsis genes)**	**Source Species**	**Lettuce Scaffold**	**Map Location in Candidate maps**	**Flanking markers from original map**	
L-iditol 2-dehydrogenase	1.1.1.14		*E. coli*	Lsat.1.v3.g.2.145	Lg2.169.053	BTNV_0	ANKZ_0
squalene monooxygenase	1.14.14.17	AT1G58440	*A. thaliana*	Lsat.1.v3.g.5.228	Lg5.127.535	BDIA_0	BHED_0
flavanoid 3′,5′-hydroxylase	1.14.13.88		*Glycine max*	Lsat.1.v3.g.5.3110	Lg5.156.565	BZCN_0	BJDZ_0
sucrose-phosphotase-	3.1.3.11	AT4G10120	*A. thaliana*	>Lsat.1.v3.g.6.288	Lg6.151.862	BHQG_0	BYQK_1
cellulose synthase 6	2.4.1.12	AT5G64740	*A. thaliana*	Lsat.1.v3.g.6.2566	Lg6.137.497	ABDX_0	BCON_0
cellulose synthase A	2.4.1.12	AT5G44030	*A. thaliana*	Lsat.1.v3.g.9.619	Lg9.3.895	ANJU_0	ANAJ_0

### 3.3 Phenotype variation within the mapping population

A PCA model built on the full spectral profiles of the progeny populations showed separation of the controlled environment trial from the two field trials along the second principal component (PC) (capturing 14.5 % of the variation) (Fig. 1B). From the loadings for PC2 (Fig. 1D), the metabolite features explaining this separation were higher amounts of sucrose, inulin, and glucose in the controlled condition and lower amounts of branched chain amino acids, succinate, choline and malic acid compared to the residual/high nitrogen trials. Across the mapping population sucrose variation was highest in the controlled environment (a factor of 4 times more concentrated in RIL 57, the most abundant in sucrose, compared to the least concentrated, RIL 61. Variation was lowest in the high nitrogen trial where RIL 56 was 2.7 times higher than RIL 26 (Supplementary Table S3). By contrast glucose variability was highly plastic in the field trials. Variance was lowest in the controlled environment where the parental genotypes *L. sativa* and *L. serriola* were highest and lowest respectively and no transgressive segregation was observed (Supplementary Fig. S2). Most variance was observed in the residual nitrogen trial, where glucose concentration was over four times higher in RIL 58 than low glucose RIL 3 which had the lowest glucose content of any RIL across all trials. Glucose abundance was consistently low in the wild parent *L. serriola* and the lowest across the population in the controlled environment trial. Inulin was functionally absent in the residual nitrogen trial for some RILs, and was on average nine times lower than either other trial. Only RIL 123 accumulated higher inulin in these conditions, over double the next highest of RIL 15.

Genotypic segregation was observed across the metabolome. Parental lines typically represented extreme phenotypes in the context of the mapping population, although varying degrees of transgressive segregation was observed for most metabolites, particularly in the field trials (select data are shown in Supplementary Fig. S2). The controlled environment gave rise to the greatest spread of variation across the population. This indicates genetic plasticity across the population. ANOVAs performed on log_10_ transformed data showed that there was significant genetic variability for all the chemotyping traits (data not shown; p < 0.05), indicating that ^1^H NMR derived datasets were suitable for further QTL analysis.

### 3.4 QTL mapping

**Fig. 4:**
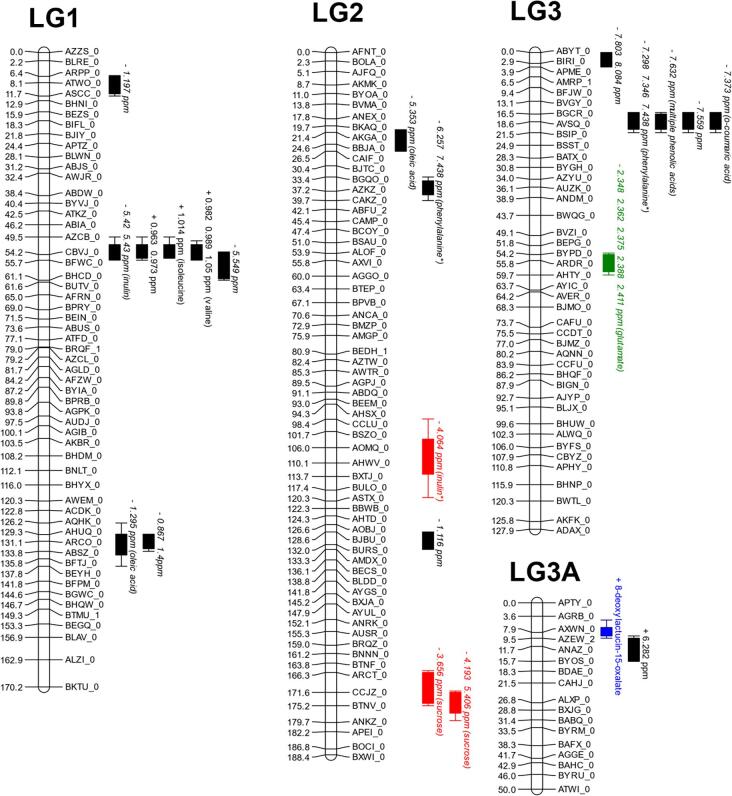

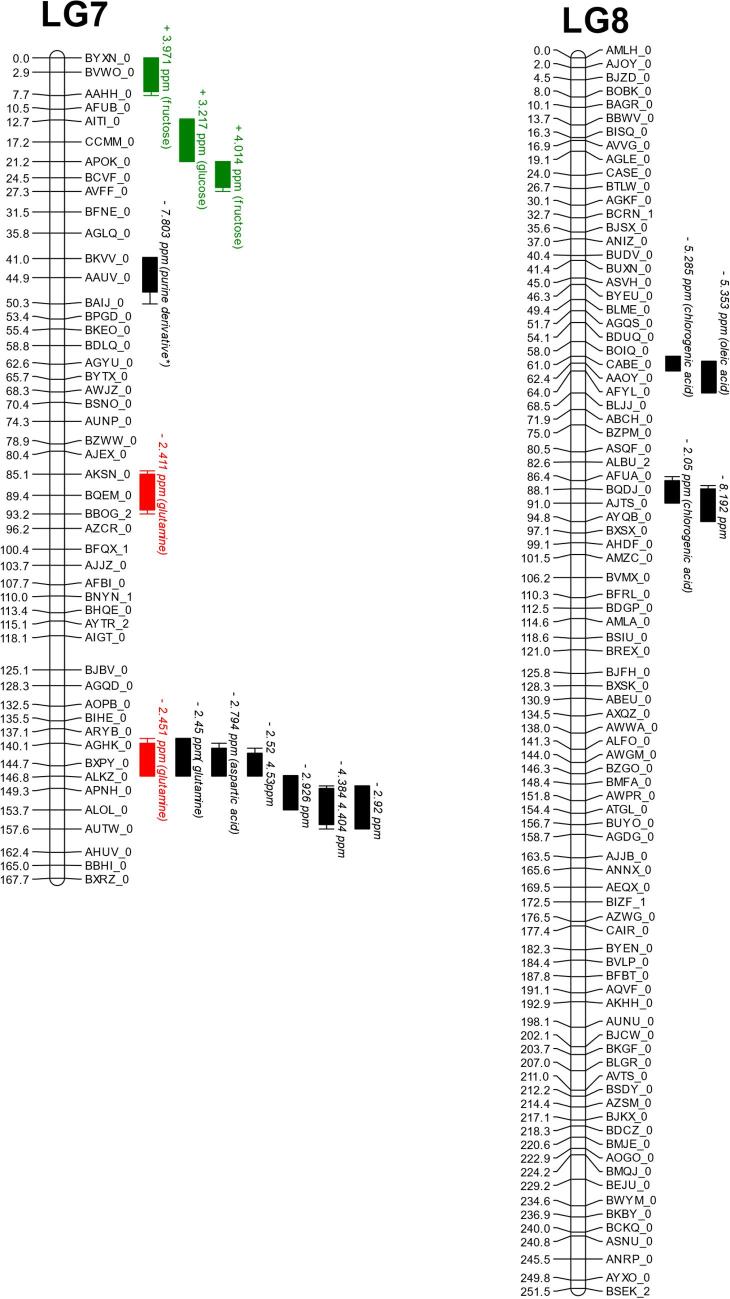

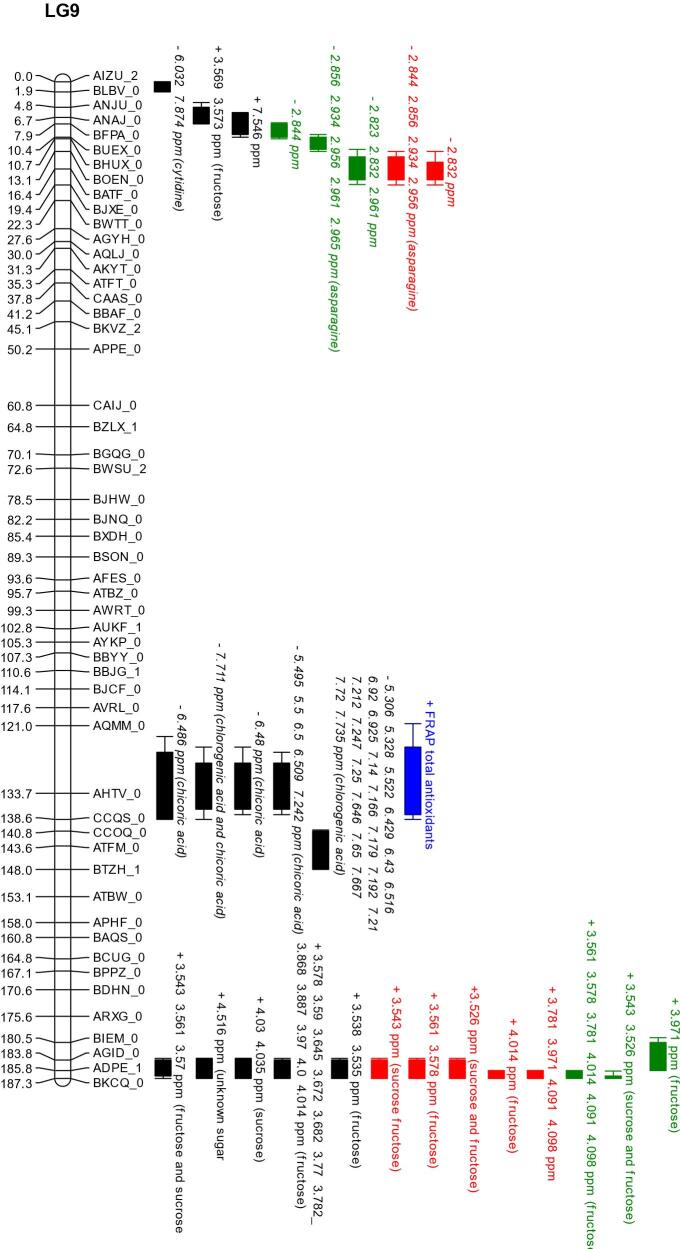
QTL map showing all markers. Red bars represent traits within the high nitrogen trial. Green bars represent traits within the low nitrogen trial. Black bars represent traits within the controlled environment trial. Blue bars represent traits within the controlled environment trial which were not identified by NMR but by the appropriate assay. QTL driven by *L. sativa* are shown as bars labelled with plus (+) symbols before the name and those driven by the wild parent *L. serriola* are shown with a minus (−). Map positions are given in cM, listed to the right of each linkage group. QTL are listed by the ^1^H NMR peak ppm, and where applicable the identity. Where multiple peaks co-locate exactly they are listed on the same bar; these are typically resultant from multiple NMR peaks corresponding to a single compound. Bars represent 1 LOD interval, with the whiskers representing a 2 LOD interval.

A total of 187 QTL were obtained. Of these, 63 unique QTL were associated to 29 identifiable compounds plus total antioxidant activity. QTL hotspots appear on chromosomes 4, 7 and 9 (Table 1 and Fig. 4).

The co-locating QTL for fructose on linkage group (LG) 9 at 185 cM was identified in all three growing environments, accounting for 22 % (high nitrogen) to 36 % (controlled environment) of the total variation for this trait. Sucrose also maps to the same locus, implying a genetic control early in sugar metabolism underlying this region. The glucose QTL at linkage group 4 at 165 cM, which is present from the high nitrogen trial and is driven by the *L. serriola* allele, co-locates with a QTL driven by *L. sativa* from the controlled environment. An additional QTL for glucose, with a higher concentration associated with the *L. serriola* allele, appears on linkage group 5 at 155 cM in both high and low nitrogen field trials, accounting for 14.8 % and 15.9 % of the population variation respectively. Interestingly, this locus co-located with a QTL for 15-p-hydroxyphenylacetyllactucin-8-sulfate derived from NMR data and which also was driven by the *L. serriola* allele and for multiple phenolic acids in the high nitrogen trial. Additional QTL for 8-deoxylactucin-15-oxalate and 15-p-hydroxyphenylacetyllactucin-8-sulfate were identified from HPLC data, on linkage groups 3A and 4 respectively. These accounted for 23–31 % of variance in the population.

A QTL for total antioxidant activity, accounting for 15.8 % of population variation, co-locates to the C9 133 cM chicoric acid and is close to a chlorogenic acid QTL at 143 cM, implying this region is important for the accumulation of these antioxidant molecules in wild lettuce. The chicoric acid QTL on C9 accounts for 21.2 % of variation and the chlorogenic acid QTL locates to 143 cM on LG9 accounting for 30.3 % of variation. In each case the was a higher concentration of metabolite in *L. serriola*. The controlled environment revealed the most QTL.

### 3.5 Identification and mapping of candidate genes

A total of 403 distinct protein sequences were extracted from KEGG and reverse translated into transcriptome sequences. Sequences related to sugar (293), polyphenolics (44), and sesquiterpene synthesis (64) pathways were screened, with 136 sugar, 49 polyphenolic and 51 STLs assigned an identity and by using BLAST, matched to scaffolds of the v3 lettuce genome (Truco et al., 2013). These were added to the existing marker map using Kyazma Join Map 4 software to identify possible co-location with existing QTL.

Six genetic sequences were identified which aligned with related QTL (Table 2). Of particular interest were a sequence for squalene monooxygenase, on Lsat.1.v3.g.5.228; a gene which catalyses the oxidation of the triterpenoid squalene (EC 1.14.14.17), mapped to linkage group 5 at 156 cM. This co-located with the only sesquiterpene QTL generated from NMR, that for 15*-p-*hydroxyphenylacetyllactucin-15-sulphate from the low N population (NMR peak 6.835 ppm).

After quality checks 293 sugar sequences, 136 SNP containing sequences were added to the marker map. A sequence from *Arabidopsis* identified as L-iditol 2-dehydrogenase (Last.1.v3.g.2.145 (EC 1.1.1.14)), involved in metabolism of various sugars, mapped to linkage group 2 at 169 cM, to the same location as a QTL for sucrose (Fig. 4.) indicating that this is a potential dehydrogenase enzyme responsible for sucrose variability in the mapping population. This QTL was identified in high N conditions, and showed higher levels of the compound were driven by the *L. serriola* genotype and could be a way to increase palatability in domesticated lettuces.

A candidate gene mapping to the same location as a relevant QTL was for sucrose-phosphate synthase (EC 2.4.1.14), which synthesises sucrose-6-phosphate from UDP-glucose. This mapped to linkage group 6 at 152 cM and to a QTL derived from NMR peak 3.543 which was represents both a sucrose and a fructose peak overlapping. This QTL was responsible for increased sucrose/fructose with the *L. sativa* genotype and was only identified within the controlled environment.

Additionally, a gene sequence derived from the protein sequences of a cellulose synthase (EC 2.4.1.12), which converts fructose to cellulose, mapped to linkage group 9 at 3.9 cM. This co-located to fructose QTL (NMR peaks 3.573 ppm and 3.538 ppm) from the controlled environment which resulted in higher concentrations of fructose in *L. sativa*.

A sequence identified as flavonoid 3′,5′-hydroxylase (EC 1.14.13.88), by ourselves and Zhang (2018) mapped to linkage group 5 at 152 cM. This enzymatic class is involved in several locations within the flavonoid metabolism pathway (Kanehisa and Goto, 2000), including anthocyanin and phenolic acid production. The sequence underlies QTL for multiple phenolic acids (NMR peaks 7.253 ppm, 6.425 ppm and 6.452 ppm; Supplementary Table S2). However as these peaks represent protons in the phenolic backbone, it was not possible to identify which individual metabolite(s) are represented. This QTL was found the low N population only, and showed higher levels of the compound were driven by the *L. serriola* genotype. This was the only candidate gene found to underlie our QTL for phenolic compounds. QTL analysis of the population identified three genetic hotspots for phenolic traits, one on linkage group two at around 133 cM (putative caffeoyl-CoA methyltransferase, Lsat.1.v3.g.2.1786), and on linkage group 4 at around 50 cM (Flavone synthase, Lsat.1.v3.g.4.815), while the third includes the flavonoid 3′,5′-hydroxylase candidate gene locus on linkage group 5 centred about 145 cM (Lsat.1.v3.g.5.3110) (Supplementary Table S2).

## 4 Discussion

### 4.1 Variation between population parents

The main differences between the parental genotypes was found to be a substantial change in the concentration of sugars, especially with regards to fructose, which is has the highest relative sweetness of the sugars (Pangborn, 1963). This is a consequence of conventional breeding techniques being used to select more palatable lettuce from the wild progenitor and increase fructose content by 9 x in the domesticated genotype. Phenolics, however, have selected against during the domestication process, typically on the basis of their acidic and bitter flavour (Drewnowski and Gomez-Carneros, 2000) and lack of a clear phenotypic benefit to conventional breeders. Modern consumers are more aware of the health benefits of eating vegetables, which polyphenols help to provide (Spencer, 2009, Mubarak et al., 2012, Lima et al., 2014, Fraga et al., 2019, Aravind et al., 2021) and so it is possible that, as breeding programmes develop to reflect consumer desire for enhanced nutritional traits in their food crops, the loci and markers associated with phenolic compounds will become commercially important.

Sub-optimal supply of nitrogen caused a strong trend for the domesticated lettuce phenotype to converge with that of the wild type, resulting in a narrower range of concentrations for each metabolite studied. The domesticated parent appears to be more affected by the nitrogen availability than *L. serriola*, as the metabolic profile of *L. sativa* is substantially changed between high and low nitrogen environments, in contrast to the wild parent which showed very little impact of growing in different concentrations of nitrogen. From this we can infer that the domesticated plant is better adapted to exploit commercial growing conditions, but retains some plasticity when challenged with adverse conditions. The difference between the chemotype profile seen in the controlled environment compared to field conditions in both species highlights the usefulness of utilising multiple trial conditions to promote identification of breeding markers that are stable in different growing environments.

### 4.2 Variation between environments

Differences in absolute amounts of a metabolite between genotypes frequently contrasted with the capacity of a genotype to display phenotypic plasticity in response to different growing environments. We observed greater sugar accumulation in *L. serriola* when it was exposed to the field conditions in comparison to the controlled environment, notably fructose content was increased by nearly 6.5x in the low N trial. Glucose and sucrose concentrations, while higher in the domesticated lettuce under most conditions, were higher in the wild parent genotype under low N. The rationale for this is not fully understood, but it may be that the wild species retains the capacity to adapt to more variable conditions or may represent that the highly domesticated lettuce has been selected for adaptation to a particular growing environment in the case of sugar accumulation. However, *L. sativa* showed the greatest plasticity across the three different environments in terms of polyphenol and pheophytin content, which were both much more abundant in field trials than in the controlled environment trial, indicating greater level of environmental adaptability. These data indicate that the domesticated plant is still capable of reverting back toward a more resilient phenotype when environmental pressure makes this beneficial. The low N trial also led to much higher concentrations of the phenolic acids, which act as antioxidants and herbivory defence molecules in plants and therefore are produced under stress, in both parental genotypes (Treutter, 2006, Skłodowska et al., 2011, Mithöfer and Boland, 2012).

### 4.3 Phenotype variation across the mapping population

PCA analysis of the populations showed that there is a great deal of overlap between the two field grown trials, despite extreme changes in nitrogen application, while the controlled environment clustered separately. This is a consequence of the field trials exposing the plants to conditions that are more similar to each other than to the controlled environment. Variation within the trial populations as a result of genetic variation shows a range of chemotypes largely within the range of the parental chemotypes, with some variation extending beyond this as the result of unmasked genes. This concept of transgressive segregation is critical for the breeding of beneficial traits into new cultivars as it relies on adding genetic information from the wild parent. The data presented in this paper shows that there is the genetic potential to identify QTL and associated markers which can be used as the basis for breeding more extreme beneficial traits into the already heavily domesticated *L. sativa* cultivar. Some peaks appear to be linked without any known relationship between the compounds, for example linkage group 5 at 155–160 cM there are QTL for glucose, STLs and phenolics. This may result in genetic linkage, therefore it is useful to see correlations between concentrations of functionally unrelated compounds in terms of breeding as this may cause undesirable phenotypes to be inherited with the beneficial trait.

### 4.4 Metabolic Characteristics

Zhang et al. (2007) suggested that hotspots relating to pre and postharvest quality traits may be due to related metabolic regulation. The present analysis has shown that groups of metabolites linked to the synthesis or regulation of the same class of compound cluster together in hotspots. We have observed this with the clustering of phenolic acid QTL and with the clustering of sugar QTL, both on linkage group 9. This indicated that the locus harbours a gene or genes regulating early stages of the metabolic pathway, prior to its divergence into the synthesis or catabolism of individual metabolites within the broader group. This population represents a wild parent, and a domesticated parent which has previously undergone significant breeding for agricultural traits and consumer traits. It is therefore understandable that some regions contain more QTL than others. Clusters were identified on LG1, LG3, LG4 and LG7 and LG9. Clustering of QTL is important with regards to breeding due to pleiotropic effects in these regions. Ideally adoption of a marker underlying a QTL cluster of interest could lead to the adoption of a number of desirable effects (Li et al., 2022). Finemapping opens up the potential to identify genes underlying QTL, improving our understanding of the mechanism of controlling absolute concentrations of compounds in a species.

### 4.5 Metabolite QTL

A number of QTL were found relating to nutritional and taste traits. QTL for sugars, and bitter sesquiterpene lactones have been identified. Additionally, we identified QTL for numerous acids, which can increase perceived sweetness through mixing interactions with bitter tasting compounds by complementing sweetness(Drewnowski, 2001) were discovered. A QTL for FRAP antioxidant potential was found, which co-locates to QTL for chicoric acid, a phenolic acid and known antioxidant. The primary location for all these traits is on LG 9 in two major hotspots, around 135 cM and 185 cM. We identified two QTL regions where the wild parental allele caused higher levels of glucose (Lg 5 155 cM and Lg 4 167 cM), accounting for around 15 % of the phenotypic variation within the population each. Each of these regions presents potential to increase sugars and improve the taste of existing lettuce cultivars, while increasing phenolic acid levels may compliment the sweetness of commercial cultivars while providing a good source of antioxidants, which are related to improved health (Selma et al., 2009, Wootton-Beard and Ryan, 2011, Del Rio et al., 2013). Preferential breeding for sugar content as a major factor of taste has caused a higher sugar content in the *L. sativa* parent when compared to the undomesticated *L. serriola* parent. By comparison, phenolic acid and fatty acid concentration was observed to be higher in the *L. serriola* genotype, indicating that these compounds may have been selected against. Amino acid concentration varied between amino acids as to which parent contributed more abundance of a particular amino acid. Phenylalanine, leucine, glutamine, and aspartate concentrations were higher in the wild parent, while accumulation of valine, and isoleucine was associated with the domesticated genotype. It is unclear how this change in amino acid profile affects the ability of the plants to tolerate different conditions, though some polyphenols are derived from amino acids via the shikimate pathway (Hisaminato et al., 2001). Hence there may be differences in the ability of a plant to synthesis secondary metabolites such as beneficial phenolic from the amino acid precursors, particularly in nitrogen limited conditions.

Of particular importance to breeding goals was the hotspot in the low nitrogen trial at linkage group 5, at 155 cM. This region contained a series of QTL driven by the *L. serriola* allele. QTL located here were for glucose, multiple phenolic acids, including and chlorogenic acid and 15*-p-*hydroxyphenylacetyllactucin-15-oxalate. The STL 15*-p-*hydroxyphenylacetyllactucin-15-oxalate is not believed to make any contribution to taste perception and is one of the few non-bitter STLs (Chadwick et al., 2016). The combination of a glucose QTL and an STL QTL which is not believed to affect taste is of great utility, especially as candidate genes underlying these QTL were identified. These findings offer a clear opportunities for breeders to use marker assisted breeding to generate novel cultivars which taste better and which have enhanced nutritive qualities compared to the Salinas cultivar from which the mapping population used in this study was derived.

### 4.6 QTL x environment Interaction

Three environments were investigated; an indoor controlled growth chamber, and two field trials grown simultaneously where plots were either high or low nitrogen. One major QTL hotspot for fructose at linkage group 9, which also influences sucrose, remained stable in all environments and which is therefore a useful region from which to develop breeding markers for this trait. Other QTL for sugars, and also QTL for phenolic acids were stable over two of the three trials. Of the QTL regions most were not stable across the environments, implying a strong environmental factor was influencing most traits. This can also be seen from the metabolomics data, with broad ranging differences in metabolite profile across the different environments.

120 QTL, approximately two thirds of the total found in this study, were identified from the controlled environment where biotic and abiotic stresses were minimised. As the phenotypic variation was most limited under the stress of the low nitrogen condition this environment gave rise to the least QTL due to the environmental factors taking precedence over genetic factors.

### 4.7 Associating candidate genes with existing marker map

Candidate gene analysis using potential genes of interest directly as markers has utility for rapidly identifying the genes which underlie QTL, by highlighting sequences under the QTL which encode enzymes which are involved in synthesis of such molecules.

Mapping candidate genes in this way will expose the stages of metabolic pathways which have the greatest regulatory impact on final concentration of metabolites within the mapping population, provide powerful potential markers for plant breeders, and lead to better understanding of metabolite biosynthesis. Identifying strong candidates can speed up breeding and help us to understand the pathways being tailored. In the scope of this study we were unable to identify all genes in the region, relying on information from characterised biochemical pathways, and consequently were not able to identify all genes underlying some major loci.

Several of the major QTL hotspots such as that of fructose at LG9 183 cM, chicoric acid on LG9 133 cM and chlorogenic acid at LG9 143 cM did not have candidate genes associated with them. The genes which underlie these loci may be involved in regulation of the major synthesis pathway, with enzyme modulators accounting for only 5 % of the total number of genes (Mi et al., 2013), and a series of other regulators, promoters, and transcription factors may instead lead to the segregation within the population being derived from this locus. RNAseq offers the opportunity to investigate genetic regulators outside the transcription of enzymes involved in metabolite biosynthesis. This technique was nonetheless useful to identify genes of interest underlying some QTL.

### 4.8 Mapping of sugar synthesis genes

The most significant fructose QTL was observed in all three populations on linkage group 9, 183 cM. However, this did not co-locate to any of the candidate genes identified. We can be confident that the location of the QTL is correct owing to its high LOD score and stability across all populations and differing analysis techniques. The sugar candidate genes which did map underneath the QTL were for sucrose-phosphate synthase, cellulose synthase, fructose-1,6-bisphosphatase I, and L-iditol 2-dehydrogenase. In the case of L-iditol 2-dehydrogenase and fructose bisphosphatase aldolase I, these genes both mapped under sucrose QTL on linkage group 2, L-iditol 2-dehydrogenase is an oxidoreductose, which converts sorbitol to fructose, is involved in the metabolism of other pentoses (Kanehisa and Goto, 2000). Fructose-1,6 −bisphosphatase and fructose-2,6 −bisphosphatase have previously been implicated in sucrose metabolism (Rufty and Huber, 1983, Stitt and Heldt, 1985, Cho et al., 2012), despite ostentatiously being involved in fructose metabolism; therefore this appears to be a suitable candidate for the driving segregation between the types of sugars at this locus. It is interesting to note that in this population the wild type, which could be expected to have a lower sucrose content due to selective breeding of the domesticated variety favouring sugar production, was instead driving the increase in sucrose concentration at this locus. This can be accounted for understanding that this gene is responsible for converting sucrose to starch, and therefore it is likely that the higher sucrose concentration is a result of lower starch stores in the wild type, driven by a selective pressure for domesticated plants to keep greater store of starch.

Other candidate genes were the sucrose-phosphate synthase which co-locates to the QTL of a peak representing both fructose and sucrose, on linkage group 6, 158 cM, and cellulose synthase on linkage group 9 3.9 cM which is a glycosyltransferase involved in generating cellulose from UDP-glucose. While the cellulose synthase gene co-locates to a QTL for fructose rather than glucose, there may be a similar modification in the fructose pathway.

### 4.9 Mapping of flavonoid synthesis genes

The only flavonoid candidate gene sequence to map to a QTL was the oxidoreductase flavonoid 3′,5′-hydroxylase. This is involved in a number of reactions with nine separate occurrences shown in KEGG pathway, and co-locates to three peaks representing a proton present in multiple phenolics, one for chlorogenic acid and another for caftaric acid. This appears to be a very likely candidate for the gene underlying the QTL as it should be expected to catalyse modifications in a range of compounds, and this was observed in the QTL hotspot. Similarly to the major fructose QTL on linkage group 9, neither of the two linkage group 9 QTL for chicoric acid and chlorogenic acid co-located to any of the candidate genes and, therefore we reason that this is most likely the result of a regulatory gene in this location rather than an enzymatic regulatory step directly involved in metabolism of the phenolic acids. Other genes involved in flavonoid biosynthesis spread across all chromosomes, with highly concentrated regions on linkage group 2 at 113 cM, LG4 at 50 cM, LG5 at 90 cM, which imply heavy co-inheritance of genes at these locations. However, none of these were responsible for segregation within our mapping population.

### 4.10 Mapping of sesquiterpenoid synthesis genes

One candidate gene sequence mapped to a QTL for a sesquiterpenoid. This was the squalene monooxygenase gene sequence AT1G58440 which mapped to NMR peak 6.835 in the low nitrogen trial. This peak was attributed to the sesquiterpene lactone 15*-p-*hydroxyphenylacetyllactucin-8-sulphate. Squalene monooxygenase is an oxidoreductase thought to be a rate limiting step in sterol biosynthesis in which it is involved (Ma et al., 2007). Squalene is a triterpenoid in a separate pathway to sesquiterpene but also derived from farnesyl pyrophosphate, a precursor to sesquiterpene backbone synthesis. This appears to be a very likely candidate for the gene underlying the QTL as it could be expected to catalyse modifications in a range of terpenoids including those found in lettuce, and while squalene is not present in lettuce, a sesquiterpenoid oxidoreductase would likely have high homology to this triterpenoid oxidoreductase. A sesquiterpenoid oxidoreductase with high homology to squalene monooxygenase is likely the gene influencing this QTL. Only two other QTL for sesquiterpene lactones could be identified in the population, both from HPLC data derived from the controlled environment trial but no candidate genes aligned to either of these QTL.

## 5 Conclusion

We demonstrate that NMR spectroscopy can be an effective means of returning metabolomics data for the purpose of QTL mapping. The speed and completeness of the untargeted NMR approach is sufficient to generate robust QTL for compound classes, if not specific compounds. This approach allows QTL mapping of chemotypes applicable to many fields of plant research. We were able to use this method to identify range of QTL without losing the fidelity necessary to direct effective plant breeding.

We identified a significant QTL for fructose on chromosome 9 which was present across all growing conditions, co-locating with a QTL for polyphenols. This QTL accounted for 36 % of variation in fructose across our population, all identified QTL in this population can account for 80 % of the variance in the controlled environment. These QTL have use as breeding targets to generate lettuce varieties which have a sweet taste that appeals to consumer preferences, while maintaining net nutritional benefit. The scope of this study demonstrates that these QTL were stable in response to high and residual nitrogen fertilisation in a UK summer climate, and in controlled environments. QTL stability could not be established for more diverse growing conditions such as in major production areas in Andalucia, Spain and California, USA, nor under novel light programs of modern vertical farming.

We also identified a QTL and candidate gene regulating the sesquiterpene lactone 15-p-hydroxyphenylacetyllactucin-8-sulphate on linkage group 5. This is of particular importance as it has previously been demonstrated to have a lower bitter taste threshold than other sesquiterpene lactones, and represents a suitable target to incorporate into plant varieties to maintain plant defence and health functionality without adversely affecting flavour. This paper provides novel insight into the regulation of key quality traits relating to taste and nutrition in lettuce, with clear candidate genes and breeding targets identified that will enable the genetic improvement of lettuce for the benefit of growers and consumer.

## Funding

This work was supported by a BBSRC Case studentship awarded to the University of Reading and Tozer Seeds (BB/G017670/1).

## CRediT authorship contribution statement

**Martin Chadwick:** Writing – original draft, Investigation, Formal analysis, Data curation. **Jonathan R. Swann:** Writing – review & editing, Supervision, Software, Data curation. **Frances Gawthrop:** Funding acquisition. **Richard Michelmore:** Software, Funding acquisition. **Davide Scaglione:** Supervision, Methodology. **Maria Jose-Truco:** Supervision. **Carol Wagstaff:** Writing – review & editing, Supervision, Funding acquisition, Conceptualization.

## Declaration of competing interest

The authors declare the following financial interests/personal relationships which may be considered as potential competing interests: Martin Chadwick reports financial support was provided by Tozer Seeds. If there are other authors, they declare that they have no known competing financial interests or personal relationships that could have appeared to influence the work reported in this paper.

## Data Availability

Data will be made available on request.

## References

[b0005] Aravind S.M., Wichienchot S., Tsao R., Ramakrishnan S., Chakkaravarthi S. (2021). Role of dietary polyphenols on gut microbiota, their metabolites and health benefits. Food Research International.

[b0010] Bischoff T.A., Kelley C.J., Karchesy Y., Laurantos M., Nguyen-Dinh P., Arefi A.G. (2004). Antimalarial activity of Lactucin and Lactucopicrin: Sesquiterpene lactones isolated from Cichorium intybus L. Journal of Ethnopharmacology.

[b0015] Brockhoff A., Behrens M., Massarotti A., Appendino G., Meyerhof W. (2007). Broad Tuning of the Human Bitter Taste Receptor hTAS2R46 to Various Sesquiterpene Lactones, Clerodane and Labdane Diterpenoids, Strychnine, and Denatonium. Journal of Agricultural and Food Chemistry.

[b0030] Catalkaya G., Venema K., Lucini L., Rocchetti G., Delmas D., Daglia M., De Filippis A., Xiao H., Quiles J.L., Xiao J. (2020). Interaction of dietary polyphenols and gut microbiota: Microbial metabolism of polyphenols, influence on the gut microbiota, and implications on host health. Food Frontiers.

[b0035] Chadwick M., Gawthrop F., Michelmore R.W., Wagstaff C., Methven L. (2016). Perception of bitterness, sweetness and liking of different genotypes of lettuce. Food Chemistry.

[b0040] Chadwick M., Trewin H., Gawthrop F., Wagstaff C. (2013). Sesquiterpenoids Lactones: Benefits to Plants and People. International Journal of Molecular Sciences.

[b0045] Chen F.E., Huang D.-B., Chen Y.-Q., Ghosh G. (1998). Crystal Structure of p50/p65 Heterodimer of Transcription Factor NF-κB Bound to DNA. Nature.

[b0050] Cho M.-H., Jang A., Bhoo S.H., Jeon J.-S., Hahn T.-R. (2012). Manipulation of triose phosphate/phosphate translocator and cytosolic fructose-1, 6-bisphosphatase, the key components in photosynthetic sucrose synthesis, enhances the source capacity of transgenic Arabidopsis plants. Photosynthesis Research.

[b0055] Cooke L. (2007). The importance of exposure for healthy eating in childhood: A review. Journal of Human Nutrition and Dietetics.

[b0060] Del Rio D., Rodriguez-Mateos A., Spencer J.P., Tognolini M., Borges G., Crozier A. (2013). Dietary phenolics in human health: Structures, bioavailability, and evidence of protective effects against chronic diseases. Antioxidants & Redox Signaling.

[b0070] Drewnowski A. (2001). The science and complexity of bitter taste. Nutrition Reviews.

[b0075] Drewnowski A., Gomez-Carneros C. (2000). Bitter taste, phytonutrients, and the consumer: A review. The American Journal of Clinical Nutrition.

[b0080] Fraga C.G., Croft K.D., Kennedy D.O., Tomás-Barberán F.A. (2019). The effects of polyphenols and other bioactives on human health. Food & Function.

[b0085] García-Macías P., Ordidge M., Vysini E., Waroonphan S., Battey N.H., Gordon M.H., Hadley P., John P., Lovegrove J.A., Wagstaffe A. (2007). Changes in the Flavonoid and Phenolic Acid Contents and Antioxidant Activity of Red Leaf Lettuce (Lollo Rosso) Due to Cultivation under Plastic Films Varying in Ultraviolet Transparency. Journal of Agricultural and Food Chemistry.

[b0090] García C.J., Beltrán D., Tomás-Barberán F.A. (2020). Human gut microbiota metabolism of dietary sesquiterpene lactones: Untargeted metabolomics study of lactucopicrin and lactucin conversion in vitro and in vivo. Molecular Nutrition & Food Research.

[b0095] Gent M.P. (2012). Composition of hydroponic lettuce: Effect of time of day, plant size, and season. Journal of the Science of Food and Agriculture.

[b0100] Green B.G., Lim J., Osterhoff F., Blacher K., Nachtigal D. (2010). Taste mixture interactions: Suppression, additivity, and the predominance of sweetness. Physiology & Behavior.

[b0105] Hehner S.P., Heinrich M., Bork P.M., Vogt M., Ratter F., Lehmann V., Schulze-Osthoff K., Droge W., Schmitz M.L. (1998). Sesquiterpene Lactones Specifically Inhibit Activation of NF-κB by Preventing the Degradation of IκB-α and IκB-β. Journal of Biological Chemistry.

[b0110] Heimler D., Vignolini P., Arfaioli P., Isolani L., Romani A. (2012). Conventional, organic and biodynamic farming: Differences in polyphenol content and antioxidant activity of Batavia lettuce. Journal of the Science of Food and Agriculture.

[b0115] Heinrich M., Robles M., West J.E., Ortiz de Montellano B.R., Rodriguez E. (1998). Ethnopharmacology of Mexican Asteraceae (Compositae). Annual Reviews in Pharmacology and Toxicology.

[b0120] Hisaminato H., Murata M., Homma S. (2001). Relationship between the enzymatic browning and phenylalanine ammonia-lyase activity of cut lettuce, and the prevention of browning by inhibitors of polyphenol biosynthesis. Bioscience, Biotechnology, and Biochemistry.

[b0125] Kanehisa M., Goto S. (2000). KEGG: Kyoto encyclopedia of genes and genomes. Nucleic Acids Research.

[b0130] Kemboi D., Langat M.K., Siwe-Noundou X., Tshiwawa T., Krause R.W., Davison C., Smit C.J., de la Mare J.-A., Tembu V.J. (2022). 13-amino derivatives of dehydrocostus lactone display greatly enhanced selective toxicity against breast cancer cells and improved binding energies to protein kinases in silico. PLos One.

[b0135] Korstanje R., Paigen B. (2002). From QTL to gene: The harvest begins. Nature Genetics.

[b0140] Kupchan S.M., Eakin M.A., Thomas A.M. (1971). Tumor Inhibitors. 69. Structure-Cytotoxicity Relations Among the Sesquiterpene Lactones. Journal of Medicinal Chemistry.

[b0145] Lanzotti V., Anzano A., Grauso L., Zotti M., Sacco A., Senatore M., Moreno M., Diano M., Parente M., Esposito S. (2022). NMR metabolomics and chemometrics of lettuce, Lactuca sativa L., under different foliar organic fertilization treatments. Plants.

[b0150] Li J., Tu W., Xiao G., Liu T., Chen H., Tao W., Nie B., Song B. (2022). Pleiotropic QTL Underlying the Dormancy and Reducing Sugar Content in Potato Tubers Uncovered by Conditional QTL Analysis. Potato Research.

[b0155] Lima, G. P. P., F. Vianello, C. R. Corrêa, R. A. d. S. Campos and M. G. Borguini (2014). “Polyphenols in fruits and vegetables and its effect on human health.” Food and Nutrition sciences: 1065-1082.

[b0160] Llorach R., Tomás-Barberán F.A., Ferreres F. (2004). Lettuce and Chicory Byproducts as a Source of Antioxidant Phenolic Extracts. Journal of Agricultural and Food Chemistry.

[b0165] Lyß G., Knorre A., Schmidt T.J., Pahl H.L., Merfort I. (1998). The Anti-inflammatory Sesquiterpene Lactone Helenalin Inhibits the Transcription Factor NF-κB by Directly Targeting p65. Journal of Biological Chemistry.

[b0170] Ma J., Dempsey A.A., Stamatiou D., Marshall K.W., Liew C.-C. (2007). Identifying leukocyte gene expression patterns associated with plasma lipid levels in human subjects. Atherosclerosis.

[b0175] Machado, P. P., E. M. Steele, M. L. d. C. Louzada, R. B. Levy, A. Rangan, J. Woods, T. Gill, G. Scrinis and C. A. Monteiro (2020). “Ultra-processed food consumption drives excessive free sugar intake among all age groups in Australia.” European Journal of Nutrition **59**(6): 2783-2792. 10.1007/s00394-019-02125-y 31676952

[b0180] Macías F.A., Torres A., Molinllo J.G., Varela R.M., Castellano D. (1996). Potential Allelopathic Sesquiterpene Lactones from Sunflower Leaves. Phytochemistry.

[b0185] MAFF, U. (2000). Fertiliser recommendations for agricultural and horticultural crops (RB209), The Stationery Office London.

[b0190] Mannina L., Sobolev A.P., Capitani D. (2012). Applications of NMR metabolomics to the study of foodstuffs: Truffle, kiwifruit, lettuce, and sea bass. Electrophoresis.

[b0195] Mattoo A.K., Sobolev A.P., Neelam A., Goyal R.K., Handa A.K., Segre A.L. (2006). Nuclear magnetic resonance spectroscopy-based metabolite profiling of transgenic tomato fruit engineered to accumulate spermidine and spermine reveals enhanced anabolic and nitrogen-carbon interactions. Plant Physiology.

[b0200] Mi H., Muruganujan A., Casagrande J.T., Thomas P.D. (2013). Large-scale gene function analysis with the PANTHER classification system. Nature Protocols.

[b0205] Mithöfer A., Boland W. (2012). Plant defense against herbivores: Chemical aspects. Annual Review of Plant Biology.

[b0210] Moujir L., Callies O., Sousa P.M., Sharopov F., Seca A.M. (2020). Applications of sesquiterpene lactones: A review of some potential success cases. Applied Sciences.

[b0215] Mubarak A., Bondonno C.P., Liu A.H., Considine M.J., Rich L., Mas E., Croft K.D., Hodgson J.M. (2012). Acute Effects of Chlorogenic Acid on Nitric Oxide Status, Endothelial Function, and Blood Pressure in Healthy Volunteers: A Randomized Trial. Journal of Agricultural and Food Chemistry.

[b0220] Pangborn R. (1963). Relative Taste Intensities of Selected Sugars and Organic Acids. Journal of Food Science.

[b0225] Piccioni F., Capitani D., Zolla L., Mannina L. (2009). NMR metabolic profiling of transgenic maize with the Cry1A (b) gene. Journal of Agricultural and Food Chemistry.

[b0235] Rodrigues L., Silverio R., Costa A.R., Antunes C., Pomar C., Infante P., Conceição C., Amado F., Lamy E. (2020). Taste sensitivity and lifestyle are associated with food preferences and BMI in children. International Journal of Food Sciences and Nutrition.

[b0240] Rubel Mozumder N., Lee Y.-R., Hwang K.H., Lee M.-S., Kim E.-H., Hong Y.-S. (2020). Characterization of tea leaf metabolites dependent on tea (Camellia sinensis) plant age through 1H NMR-based metabolomics. Applied Biological Chemistry.

[b0245] Rufty T.W., Huber S.C. (1983). Changes in starch formation and activities of sucrose phosphate synthase and cytoplasmic fructose-1, 6-bisphosphatase in response to source-sink alterations. Plant Physiology.

[b0250] Saksvig B.I., Gittelsohn J., Harris S.B., Hanley A.J., Valente T.W., Zinman B. (2005). A pilot school-based healthy eating and physical activity intervention improves diet, food knowledge, and self-efficacy for native Canadian children. The Journal of Nutrition.

[b0255] Schauer N., Semel Y., Roessner U., Gur A., Balbo I., Carrari F., Pleban T., Perez-Melis A., Bruedigam C., Kopka J. (2006). Comprehensive metabolic profiling and phenotyping of interspecific introgression lines for tomato improvement. Nature Biotechnology.

[b0260] Schomburg C., Schuehly W., Da Costa F.B., Klempnauer K.-H., Schmidt T.J. (2013). Natural Sesquiterpene Lactones as Inhibitors of Myb-Dependent Gene Expression: Structure-Activity Relationships. European Journal of Medicinal Chemistry.

[b0265] Selma, M. a. V., J. C. Espín and F. A. Tomás-Barberán (2009). “Interaction between phenolics and gut microbiota: role in human health.” Journal of Agricultural and Food Chemistry**57**(15): 6485-6501. 10.1021/jf902107d 19580283

[b0270] Sessa R.A., Bennett M.H., Lewis M.J., Mansfield J.W., Beale M.H. (2000). Metabolite Profiling of Sesquiterpene Lactones from Lactuca Species. Journal of Biological Chemistry.

[b0275] Shi M., Gu J., Wu H., Rauf A., Emran T.B., Khan Z., Mitra S., Aljohani A.S., Alhumaydhi F.A., Al-Awthan Y.S. (2022). Phytochemicals, nutrition, metabolism, bioavailability, and health benefits in lettuce—A comprehensive review. Antioxidants.

[b0280] Siedle B., Garcia-Pineres A.J., Murillo R., Schulte-Monting J., Castro V., Rungeler P., Klaas C.A., Da Costa F.B., Kisiel W., Merfort I. (2004). Quantitative Structure−Activity Relationship of Sesquiterpene Lactones as Inhibitors of the Transcription Factor NF-κB. Journal of Medicinal Chemistry.

[b0285] Skłodowska M., Gajewska E., Kuźniak E., Wielanek M., Mikiciński A., Sobiczewski P. (2011). Antioxidant Profile and Polyphenol Oxidase Activities in Apple Leaves after Erwinia amylovora Infection and Pretreatment with a Benzothiadiazole-type Resistance Inducer (BTH). Journal of Phytopathology.

[b0290] Slater G.S., Birney E. (2005). Automated generation of heuristics for biological sequence comparison. BMC Bioinformatics.

[b0295] Sobolev A.P., Brosio E., Gianferri R., Segre A.L. (2005). Metabolic profile of lettuce leaves by high-field NMR spectra. Magnetic Resonance in Chemistry.

[b0300] Sobolev A.P., Testone G., Santoro F., Nicolodi C., Iannelli M.A., Amato M.E., Ianniello A., Brosio E., Giannino D., Mannina L. (2010). Quality traits of conventional and transgenic lettuce (Lactuca sativa L.) at harvesting by NMR metabolic profiling. Journal of Agricultural and Food Chemistry.

[b0305] Song W., Derito C.M., Liu M.K., He X., Dong M., Liu R.H. (2010). Cellular Antioxidant Activity of Common Vegetables. Journal of Agricultural and Food Chemistry.

[b0310] Spencer J.P. (2009). Flavonoids and brain health: Multiple effects underpinned by common mechanisms. Genes & Nutrition.

[b0315] Statistica. (2023). “Most consumed vegetables in the United States in 2022, by type [Graph].” The Packer Retrieved 16/08/23, 2023, from https://www.statista.com/statistics/477484/us-most-consumed-vegetable-and-vegetable-products-by-type/.

[b0320] Stitt M., Heldt H.W. (1985). Control of photosynthetic sucrose synthesis by fructose-2, 6-bisphosphate. Planta.

[b0325] Treutter D. (2006). Significance of flavonoids in plant resistance: A review. Environmental Chemistry Letters.

[b0330] Truco, M. J., H. Ashrafi, A. Kozik, H. van Leeuwen, J. Bowers, S. R. C. Wo, K. Stoffel, H. Xu, T. Hill and A. Van Deynze (2013). “An Ultra High-Density, Transcript-Based, Genetic Map of Lettuce.” G3: Genes| Genomes| Genetics. 10.1534/g3.112.004929 PMC3618349 23550116

[b0335] van Ooijen J. (2011).

[b0340] Villa-Ruano N., Velásquez-Valle R., Zepeda-Vallejo L.G., Pérez-Hernández N., Velázquez-Ponce M., Arcos-Adame V.M., Becerra-Martínez E. (2018). 1H NMR-based metabolomic profiling for identification of metabolites in Capsicum annuum cv. mirasol infected by beet mild curly top virus (BMCTV). Food Research International.

[b0345] Wang X., Liu M., Cai G.H., Chen Y., Shi X.C., Zhang C.C., Xia B., Xie B.C., Liu H., Zhang R.X. (2020). A potential nutraceutical candidate lactucin inhibits adipogenesis through downregulation of JAK2/STAT3 signaling pathway-mediated mitotic clonal expansion. Cells.

[b0350] Wishart D.S., Jewison T., Guo A.C., Wilson M., Knox C., Liu Y., Djoumbou Y., Mandal R., Aziat F., Dong E. (2013). HMDB 3.0—The human metabolome database in 2013. Nucleic Acids Research.

[b0355] Wootton-Beard P.C., Ryan L. (2011). Improving public health?: The role of antioxidant-rich fruit and vegetable beverages. Food Research International.

[b0360] Zhang F.-H., Yan Y.-L., Wang Y., Liu Z. (2016). Lactucin induces potent anti-cancer effects in HL-60 human leukemia cancer cells by inducing apoptosis and sub-G1 cell cycle arrest. Bangladesh Journal of Pharmacology.

[b0365] Zhang, F. Z., C. Wagstaff, A. M. Rae, A. K. Sihota, C. W. Keevil, S. D. Rothwell, G. J. J. Clarkson, R. W. Michelmore, M. J. Truco, M. S. Dixon and G. Taylor (2007). “QTLs for shelf life in lettuce co-locate with those for leaf biophysical properties but not with those for leaf developmental traits.” Journal of Experimental Botany: 1433-1449. 10.1093/jxb/erm006 17347132

[b0370] Zhang Y., Xu S., Cheng Y., Peng Z., Han J. (2018). Transcriptome profiling of anthocyanin-related genes reveals effects of light intensity on anthocyanin biosynthesis in red leaf lettuce. PeerJ.

