# Supplementary material for: Mapping taste and flavour traits to genetic markers in lettuce Lactuca sativa
Source: Food Chem (Oxf). 2024 Aug 23;9:100215. doi: 10.1016/j.fochms.2024.100215 (PMC11399806; doi:10.1016/j.fochms.2024.100215)
Supplement: Supplementary Data 1 [file mmc1.docx]

# Supplementary

B

A

Lactucin-15-oxalate (m/z 348; RT = 5.1 min)

8-Deoxylactucin-15-oxalate

(m/z 332; RT = 13.3 min)

15-p-Hydroxyphenylacetyllactucin-8-sulfate

(m/z 490; RT = 15.5 min)

Oxalic

acid

Lactucopicrin-15-oxalate

(m/z 482; RT = 15.8 min)

Lactucopicrin (m/z 410; RT = 17.3 min)

Lactucin (m/z 276; RT = 7.0 min)

***Supplementary Figure S1. Identification of individual sesquiterpenoid lactone compounds using MicroTOF-Q (Q-TOF). (A)*** *Ion fragmentation patterns, (B) compound masses and retention times*

***
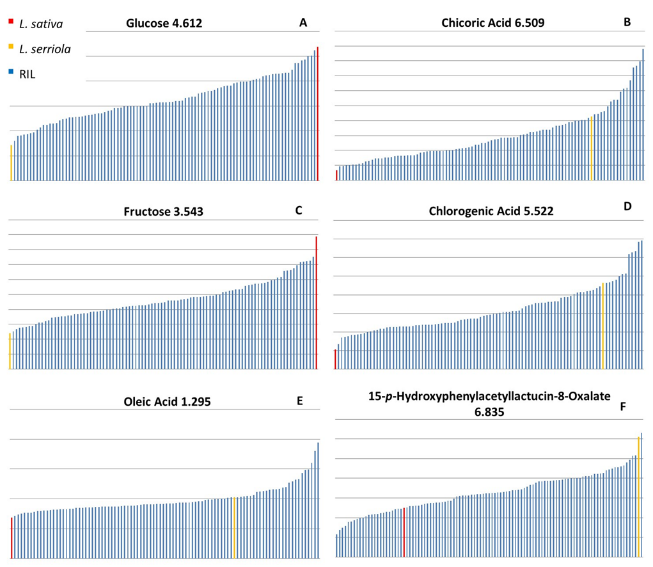
***

***Supplementary Figure S2. Segregation across population of different metabolites identified by ^1^H NMR from plants grown in the high nitrogen trial.***

*Ranking of 104 lettuce RILs (blue) and the two parental lines (red and orange) based on content of selected metabolites. A; glucose sugar, B; fructose sugar, C; chicoric acid, D; chlorogenic acid, E; oleic fatty acid; F; 15-p-hydroxyphenylacetyllactucin-15-oxalate. Analysis was by ^1^H NMR. Each bar represents a mean of samples from four independent blocks with each block replicate represented by three plants. Transgressive segregation (phenotypic variation extending beyond the limits of either parent) is shown for chicoric acid, chlorogenic acid, oleic acid and 15-p-hydroxyphenylacetyllactucin-8-oxalate indicating masked genes are present which represent potential breeding targets for these traits****.***

***Supplementary Table S1. Summary of Metabolite ^1^H Chemical Shift Identified within Lettuce Extracts.***

^1^H chemical shifts are reported with respect to TSP signal (δ = 0.0 ppm). Identities were recorded for peaks identified from ^1^H NMR spectra from both *L. sativa* and *L. serriola* parental lines. Groups where indicated are according to databases within The University of Reading. ^1^H multiplicity definitions; d = doublet, dd = doublet of doublets, ddd = doublet of doublet or doublets, m = multiplet, s = singlet, t = triplet. Peak identity was elucidated by comparison to HMDB databases, reported literature (Sessa et al., 2000; Sobolev et al., 2005), databases within The University of Reading and based on internal standards.

**Supplementary Table S2, Candidate genes which were able to be used as markers.** Sequence data from metabolic pathways related to our compounds of interest were collected, assigned an identity based on the function in the organism the sequence was derived from. Sequences were aligned to the lettuce genome and where polymorphism could be identified, these sequences were then used as markers in a specific updated marker map. Shown are the tentative name, enzyme commission identifier, and Arabidopsis TAIR identifier where appropriate, the organism the sequence was taken from, and where the marker aligned to in the draft lettuce sequence, and the marker map. Due to the mapping algorithm to recreate the map, distances are not equivalent to the original map, though the marker order has not changed.

**Supplementary Table S3, Summary values of parent and RIL population, from NMR integrations.** Relative abundance of metabolites shows a wide variation in metabolite concentration with transgressive segregation of phenotypes indicating unmasking of genes and the capacity of this population to produce effective breeding markers.

# References

Aravind, S. M., S. Wichienchot, R. Tsao, S. Ramakrishnan and S. Chakkaravarthi (2021). "Role of dietary polyphenols on gut microbiota, their metabolites and health benefits." Food Research International **142**: 110189.

Bischoff, T. A., C. J. Kelley, Y. Karchesy, M. Laurantos, P. Nguyen-Dinh and A. G. Arefi (2004). "Antimalarial activity of Lactucin and Lactucopicrin: sesquiterpene lactones isolated from Cichorium intybus L." Journal of ethnopharmacology **95**(2): 455-457.

Brockhoff, A., M. Behrens, A. Massarotti, G. Appendino and W. Meyerhof (2007). "Broad Tuning of the Human Bitter Taste Receptor hTAS2R46 to Various Sesquiterpene Lactones, Clerodane and Labdane Diterpenoids, Strychnine, and Denatonium." Journal of Agricultural and Food Chemistry **55**(15): 6236-6243.

Cankar, K., P. Bundock, R. Sevenier, S. T. Häkkinen, J. C. Hakkert, J. Beekwilder, I. M. van der Meer, M. de Both and D. Bosch (2021). "Inactivation of the germacrene A synthase genes by CRISPR/Cas9 eliminates the biosynthesis of sesquiterpene lactones in Cichorium intybus L." Plant Biotechnology Journal **19**(12): 2442-2453.

Cankar, K., J. C. Hakkert, R. Sevenier, E. Campo, B. Schipper, C. Papastolopoulou, K. Vahabi, A. Tissier, P. Bundock and D. Bosch (2022). "CRISPR/Cas9 targeted inactivation of the kauniolide synthase in chicory results in accumulation of costunolide and its conjugates in taproots." Frontiers in Plant Science: 2920.

Catalkaya, G., K. Venema, L. Lucini, G. Rocchetti, D. Delmas, M. Daglia, A. De Filippis, H. Xiao, J. L. Quiles and J. Xiao (2020). "Interaction of dietary polyphenols and gut microbiota: Microbial metabolism of polyphenols, influence on the gut microbiota, and implications on host health." Food Frontiers **1**(2): 109-133.

Chadwick, M., F. Gawthrop, R. W. Michelmore, C. Wagstaff and L. Methven (2016). "Perception of bitterness, sweetness and liking of different genotypes of lettuce." Food Chemistry **197**: 66-74.

Chadwick, M., H. Trewin, F. Gawthrop and C. Wagstaff (2013). "Sesquiterpenoids Lactones: Benefits to Plants and People." International Journal of Molecular Sciences **14**(6): 12780-12805.

Chen, F. E., D.-B. Huang, Y.-Q. Chen and G. Ghosh (1998). "Crystal Structure of p50/p65 Heterodimer of Transcription Factor NF-κB Bound to DNA." Nature **391**(6665): 410-413.

Cho, M.-H., A. Jang, S. H. Bhoo, J.-S. Jeon and T.-R. Hahn (2012). "Manipulation of triose phosphate/phosphate translocator and cytosolic fructose-1, 6-bisphosphatase, the key components in photosynthetic sucrose synthesis, enhances the source capacity of transgenic Arabidopsis plants." Photosynthesis Research **111**(3): 261-268.

Cooke, L. (2007). "The importance of exposure for healthy eating in childhood: a review." Journal of Human Nutrition and Dietetics **20**(4): 294-301.

Del Rio, D., A. Rodriguez-Mateos, J. P. Spencer, M. Tognolini, G. Borges and A. Crozier (2013). "Dietary phenolics in human health: structures, bioavailability, and evidence of protective effects against chronic diseases." Antioxidants & Redox Signaling **18**(14): 1818-1892.

Dieterle, F., A. Ross, G. Schlotterbeck and H. Senn (2006). "Probabilistic quotient normalization as robust method to account for dilution of complex biological mixtures. Application in 1H NMR metabonomics." Analytical Chemistry **78**(13): 4281-4290.

Drewnowski, A. (2001). "The science and complexity of bitter taste." Nutrition reviews **59**(6): 163-169.

Drewnowski, A. and C. Gomez-Carneros (2000). "Bitter taste, phytonutrients, and the consumer: a review." The American Journal of Clinical Nutrition **72**(6): 1424-1435.

Fraga, C. G., K. D. Croft, D. O. Kennedy and F. A. Tomás-Barberán (2019). "The effects of polyphenols and other bioactives on human health." Food & Function **10**(2): 514-528.

García-Macías, P., M. Ordidge, E. Vysini, S. Waroonphan, N. H. Battey, M. H. Gordon, P. Hadley, P. John, J. A. Lovegrove and A. Wagstaffe (2007). "Changes in the Flavonoid and Phenolic Acid Contents and Antioxidant Activity of Red Leaf Lettuce (Lollo Rosso) Due to Cultivation under Plastic Films Varying in Ultraviolet Transparency." Journal of Agricultural and Food Chemistry **55**(25): 10168-10172.

García, C. J., D. Beltrán and F. A. Tomás‐Barberán (2020). "Human gut microbiota metabolism of dietary sesquiterpene lactones: Untargeted metabolomics study of lactucopicrin and lactucin conversion in vitro and in vivo." Molecular Nutrition & Food Research **64**(21): 2000619.

Gent, M. P. (2012). "Composition of hydroponic lettuce: effect of time of day, plant size, and season." Journal of the Science of Food and Agriculture **92**(3): 542-550.

Green, B. G., J. Lim, F. Osterhoff, K. Blacher and D. Nachtigal (2010). "Taste mixture interactions: suppression, additivity, and the predominance of sweetness." Physiology & Behavior **101**(5): 731-737.

Hehner, S. P., M. Heinrich, P. M. Bork, M. Vogt, F. Ratter, V. Lehmann, K. Schulze-Osthoff, W. Droge and M. L. Schmitz (1998). "Sesquiterpene Lactones Specifically Inhibit Activation of NF-κB by Preventing the Degradation of IκB-α and IκB-β." Journal of Biological Chemistry **273**(3): 1288-1297.

Heimler, D., P. Vignolini, P. Arfaioli, L. Isolani and A. Romani (2012). "Conventional, organic and biodynamic farming: differences in polyphenol content and antioxidant activity of Batavia lettuce." Journal of the Science of Food and Agriculture **92**(3): 551-556.

Heinrich, M., M. Robles, J. E. West, B. R. Ortiz de Montellano and E. Rodriguez (1998). "Ethnopharmacology of Mexican Asteraceae (Compositae)." Annual Reviews in Pharmacology and Toxicology **38**: 539-565.

Hisaminato, H., M. Murata and S. Homma (2001). "Relationship between the enzymatic browning and phenylalanine ammonia-lyase activity of cut lettuce, and the prevention of browning by inhibitors of polyphenol biosynthesis." Bioscience, Biotechnology, and Biochemistry **65**(5): 1016-1021.

Kanehisa, M. and S. Goto (2000). "KEGG: kyoto encyclopedia of genes and genomes." Nucleic Acids Research **28**(1): 27-30.

Kemboi, D., M. K. Langat, X. Siwe-Noundou, T. Tshiwawa, R. W. Krause, C. Davison, C. J. Smit, J.-A. de la Mare and V. J. Tembu (2022). "13-amino derivatives of dehydrocostus lactone display greatly enhanced selective toxicity against breast cancer cells and improved binding energies to protein kinases in silico." Plos one **17**(8): e0271389.

Korstanje, R. and B. Paigen (2002). "From QTL to gene: the harvest begins." Nature Genetics **31**(3): 235-236.

Kupchan, S. M., M. A. Eakin and A. M. Thomas (1971). "Tumor Inhibitors. 69. Structure-Cytotoxicity Relations Among the Sesquiterpene Lactones." Journal of Medicinal Chemistry **14**(12): 1147-1152.

Lanzotti, V., A. Anzano, L. Grauso, M. Zotti, A. Sacco, M. Senatore, M. Moreno, M. Diano, M. Parente and S. Esposito (2022). "NMR metabolomics and chemometrics of lettuce, Lactuca sativa L., under different foliar organic fertilization treatments." Plants **11**(16): 2164.

Li, J., W. Tu, G. Xiao, T. Liu, H. Chen, W. Tao, B. Nie and B. Song (2022). "Pleiotropic QTL Underlying the Dormancy and Reducing Sugar Content in Potato Tubers Uncovered by Conditional QTL Analysis." Potato Research: 1-15.

Lima, G. P. P., F. Vianello, C. R. Corrêa, R. A. d. S. Campos and M. G. Borguini (2014). "Polyphenols in fruits and vegetables and its effect on human health." Food and Nutrition sciences: 1065-1082.

Llorach, R., F. A. Tomás-Barberán and F. Ferreres (2004). "Lettuce and Chicory Byproducts as a Source of Antioxidant Phenolic Extracts." Journal of Agricultural and Food Chemistry **52**(16): 5109-5116.

Lyß, G., A. Knorre, T. J. Schmidt, H. L. Pahl and I. Merfort (1998). "The Anti-inflammatory Sesquiterpene Lactone Helenalin Inhibits the Transcription Factor NF-κB by Directly Targeting p65." Journal of Biological Chemistry **273**(50): 33508-33516.

Ma, J., A. A. Dempsey, D. Stamatiou, K. W. Marshall and C.-C. Liew (2007). "Identifying leukocyte gene expression patterns associated with plasma lipid levels in human subjects." Atherosclerosis **191**(1): 63-72.

Machado, P. P., E. M. Steele, M. L. d. C. Louzada, R. B. Levy, A. Rangan, J. Woods, T. Gill, G. Scrinis and C. A. Monteiro (2020). "Ultra-processed food consumption drives excessive free sugar intake among all age groups in Australia." European Journal of Nutrition **59**(6): 2783-2792.

Macías, F. A., A. Torres, J. G. Molinllo, R. M. Varela and D. Castellano (1996). "Potential Allelopathic Sesquiterpene Lactones from Sunflower Leaves." Phytochemistry **43**(6): 1205-1215.

MAFF, U. (2000). Fertiliser recommendations for agricultural and horticultural crops (RB209), The Stationery Office London.

Mannina, L., A. P. Sobolev and D. Capitani (2012). "Applications of NMR metabolomics to the study of foodstuffs: Truffle, kiwifruit, lettuce, and sea bass." Electrophoresis **33**(15): 2290-2313.

Mattoo, A. K., A. P. Sobolev, A. Neelam, R. K. Goyal, A. K. Handa and A. L. Segre (2006). "Nuclear magnetic resonance spectroscopy-based metabolite profiling of transgenic tomato fruit engineered to accumulate spermidine and spermine reveals enhanced anabolic and nitrogen-carbon interactions." Plant Physiology **142**(4): 1759-1770.

Mi, H., A. Muruganujan, J. T. Casagrande and P. D. Thomas (2013). "Large-scale gene function analysis with the PANTHER classification system." Nature Protocols **8**(8): 1551-1566.

Mithöfer, A. and W. Boland (2012). "Plant defense against herbivores: chemical aspects." Annual Review of Plant Biology **63**: 431-450.

Moujir, L., O. Callies, P. M. Sousa, F. Sharopov and A. M. Seca (2020). "Applications of sesquiterpene lactones: a review of some potential success cases." Applied Sciences **10**(9): 3001.

Mubarak, A., C. P. Bondonno, A. H. Liu, M. J. Considine, L. Rich, E. Mas, K. D. Croft and J. M. Hodgson (2012). "Acute Effects of Chlorogenic Acid on Nitric Oxide Status, Endothelial Function, and Blood Pressure in Healthy Volunteers: A Randomized Trial." Journal of Agricultural and Food Chemistry **60**(36): 9130-9136.

Pangborn, R. (1963). "Relative Taste Intensities of Selected Sugars and Organic Acids." Journal of Food Science **28**(6): 726-733.

Piccioni, F., D. Capitani, L. Zolla and L. Mannina (2009). "NMR metabolic profiling of transgenic maize with the Cry1A (b) gene." Journal of Agricultural and Food Chemistry **57**(14): 6041-6049.

Reyes-Chin-Wo, S., Z. Wang, X. Yang, A. Kozik, S. Arikit, C. Song, L. Xia, L. Froenicke, D. O. Lavelle and M.-J. Truco (2017). "Genome assembly with in vitro proximity ligation data and whole-genome triplication in lettuce." Nature Communications **8**.

Rodrigues, L., R. Silverio, A. R. Costa, C. Antunes, C. Pomar, P. Infante, C. Conceição, F. Amado and E. Lamy (2020). "Taste sensitivity and lifestyle are associated with food preferences and BMI in children." International Journal of Food Sciences and Nutrition **71**(7): 875-883.

Rubel Mozumder, N., Y.-R. Lee, K. H. Hwang, M.-S. Lee, E.-H. Kim and Y.-S. Hong (2020). "Characterization of tea leaf metabolites dependent on tea (Camellia sinensis) plant age through 1H NMR-based metabolomics." Applied Biological Chemistry **63**(1): 1-8.

Rufty, T. W. and S. C. Huber (1983). "Changes in starch formation and activities of sucrose phosphate synthase and cytoplasmic fructose-1, 6-bisphosphatase in response to source-sink alterations." Plant Physiology **72**(2): 474-480.

Saksvig, B. I., J. Gittelsohn, S. B. Harris, A. J. Hanley, T. W. Valente and B. Zinman (2005). "A pilot school-based healthy eating and physical activity intervention improves diet, food knowledge, and self-efficacy for native Canadian children." The Journal of Nutrition **135**(10): 2392-2398.

Schauer, N., Y. Semel, U. Roessner, A. Gur, I. Balbo, F. Carrari, T. Pleban, A. Perez-Melis, C. Bruedigam and J. Kopka (2006). "Comprehensive metabolic profiling and phenotyping of interspecific introgression lines for tomato improvement." Nature Biotechnology **24**(4): 447-454.

Schomburg, C., W. Schuehly, F. B. Da Costa, K.-H. Klempnauer and T. J. Schmidt (2013). "Natural Sesquiterpene Lactones as Inhibitors of Myb-Dependent Gene Expression: Structure–Activity Relationships." European Journal of Medicinal Chemistry **63**(0): 313-320.

Selma, M. a. V., J. C. Espín and F. A. Tomás-Barberán (2009). "Interaction between phenolics and gut microbiota: role in human health." Journal of Agricultural and Food Chemistry **57**(15): 6485-6501.

Sessa, R. A., M. H. Bennett, M. J. Lewis, J. W. Mansfield and M. H. Beale (2000). "Metabolite Profiling of Sesquiterpene Lactones from Lactuca Species." Journal of Biological Chemistry **275**(35): 26877-26884.

Shi, M., J. Gu, H. Wu, A. Rauf, T. B. Emran, Z. Khan, S. Mitra, A. S. Aljohani, F. A. Alhumaydhi and Y. S. Al-Awthan (2022). "Phytochemicals, nutrition, metabolism, bioavailability, and health benefits in lettuce—A comprehensive review." Antioxidants **11**(6): 1158.

Siedle, B., A. J. Garcia-Pineres, R. Murillo, J. Schulte-Monting, V. Castro, P. Rungeler, C. A. Klaas, F. B. Da Costa, W. Kisiel and I. Merfort (2004). "Quantitative Structure−Activity Relationship of Sesquiterpene Lactones as Inhibitors of the Transcription Factor NF-κB." Journal of Medicinal Chemistry **47**(24): 6042-6054.

Skłodowska, M., E. Gajewska, E. Kuźniak, M. Wielanek, A. Mikiciński and P. Sobiczewski (2011). "Antioxidant Profile and Polyphenol Oxidase Activities in Apple Leaves after Erwinia amylovora Infection and Pretreatment with a Benzothiadiazole‐type Resistance Inducer (BTH)." Journal of Phytopathology **159**(7‐8): 495-504.

Slater, G. S. and E. Birney (2005). "Automated generation of heuristics for biological sequence comparison." BMC Bioinformatics **6**(1): 31.

Sobolev, A. P., E. Brosio, R. Gianferri and A. L. Segre (2005). "Metabolic profile of lettuce leaves by high‐field NMR spectra." Magnetic Resonance in Chemistry **43**(8): 625-638.

Sobolev, A. P., G. Testone, F. Santoro, C. Nicolodi, M. A. Iannelli, M. E. Amato, A. Ianniello, E. Brosio, D. Giannino and L. Mannina (2010). "Quality traits of conventional and transgenic lettuce (Lactuca sativa L.) at harvesting by NMR metabolic profiling." Journal of Agricultural and Food Chemistry **58**(11): 6928-6936.

Song, W., C. M. Derito, M. K. Liu, X. He, M. Dong and R. H. Liu (2010). "Cellular Antioxidant Activity of Common Vegetables." Journal of Agricultural and Food Chemistry **58**(11): 6621-6629.

Spencer, J. P. (2009). "Flavonoids and brain health: multiple effects underpinned by common mechanisms." Genes & Nutrition **4**(4): 243-250.

Statistica. (2023). "Most consumed vegetables in the United States in 2022, by type [Graph]." The Packer Retrieved 16/08/23, 2023, from <https://www.statista.com/statistics/477484/us-most-consumed-vegetable-and-vegetable-products-by-type/>.

Stitt, M. and H. W. Heldt (1985). "Control of photosynthetic sucrose synthesis by fructose-2, 6-bisphosphate." Planta **164**(2): 179-188.

Treutter, D. (2006). "Significance of flavonoids in plant resistance: a review." Environmental Chemistry Letters **4**(3): 147-157.

Truco, M. J., H. Ashrafi, A. Kozik, H. van Leeuwen, J. Bowers, S. R. C. Wo, K. Stoffel, H. Xu, T. Hill and A. Van Deynze (2013). "An Ultra High-Density, Transcript-Based, Genetic Map of Lettuce." G3: Genes| Genomes| Genetics.

van Ooijen, J. (2011). MapQTL 6: software for the mapping of quantitative trait loci in experimental populations of diploid species. Wageningen, The Netherlands.

Villa-Ruano, N., R. Velásquez-Valle, L. G. Zepeda-Vallejo, N. Pérez-Hernández, M. Velázquez-Ponce, V. M. Arcos-Adame and E. Becerra-Martínez (2018). "1H NMR-based metabolomic profiling for identification of metabolites in Capsicum annuum cv. mirasol infected by beet mild curly top virus (BMCTV)." Food Research International **106**: 870-877.

Wang, X., M. Liu, G. H. Cai, Y. Chen, X. C. Shi, C. C. Zhang, B. Xia, B. C. Xie, H. Liu and R. X. Zhang (2020). "A potential nutraceutical candidate lactucin inhibits adipogenesis through downregulation of JAK2/STAT3 signaling pathway-mediated mitotic clonal expansion." Cells **9**(2): 331.

Wishart, D. S., T. Jewison, A. C. Guo, M. Wilson, C. Knox, Y. Liu, Y. Djoumbou, R. Mandal, F. Aziat and E. Dong (2013). "HMDB 3.0—The human metabolome database in 2013." Nucleic acids research **41**(D1): D801-D807.

Wootton-Beard, P. C. and L. Ryan (2011). "Improving public health?: The role of antioxidant-rich fruit and vegetable beverages." Food Research International **44**(10): 3135-3148.

Zhang, F.-H., Y.-L. Yan, Y. Wang and Z. Liu (2016). "Lactucin induces potent anti-cancer effects in HL-60 human leukemia cancer cells by inducing apoptosis and sub-G1 cell cycle arrest." Bangladesh Journal of Pharmacology **11**(2): 478-484.

Zhang, F. Z., C. Wagstaff, A. M. Rae, A. K. Sihota, C. W. Keevil, S. D. Rothwell, G. J. J. Clarkson, R. W. Michelmore, M. J. Truco, M. S. Dixon and G. Taylor (2007). "QTLs for shelf life in lettuce co-locate with those for leaf biophysical properties but not with those for leaf developmental traits." Journal of Experimental Botany: 1433-1449.

Zhang, Y., S. Xu, Y. Cheng, Z. Peng and J. Han (2018). "Transcriptome profiling of anthocyanin-related genes reveals effects of light intensity on anthocyanin biosynthesis in red leaf lettuce." PeerJ **6**: e4607.
